# IL-13RA2 downregulation in fibroblasts promotes keloid fibrosis via JAK/STAT6 activation

**DOI:** 10.1172/jci.insight.157091

**Published:** 2023-03-22

**Authors:** Hua Chao, Lisheng Zheng, Pojui Hsu, Jinyun He, Ridong Wu, Shuqia Xu, Ruixi Zeng, Yuan Zhou, Huisi Ma, Haibo Liu, Qing Tang

**Affiliations:** 1 Division of Plastic and Reconstructive Surgery, First Affiliated Hospital of Sun Yat-sen University, Guangzhou, China.; 2 Department of Pathology, Guangdong Provincial People’s Hospital (Guangdong Academy of Medical Sciences), Southern Medical University, Guangzhou, China.; 3 Division of Vascular Surgery, the First Affiliated Hospital of Sun Yat-sen University, Guangzhou, China.; 4 Department of Pathology, Foshan Women and Children’s Hospital, Foshan, China.

**Keywords:** Dermatology, Inflammation, Fibrosis, Skin

## Abstract

Keloids are considered the manifestation of a fibroproliferative disease characterized by chronic inflammation that is induced following skin injury. Deciphering the underlying mechanism of keloid formation is essential for improving treatment outcomes. Here, we found that more macrophages were activated toward the M2 subtype in keloid dermis when compared with normal dermis. Western blotting revealed that the level of phosphorylated STAT6 (p-STAT6), a known inducer of M2 polarization, was higher in keloid fibroblasts as opposed to fibroblasts from normal dermis. Moreover, keloid fibrosis was shown to be positively correlated with the level of p-STAT6. Further, we identified downregulation of IL-13RA2, a decoy receptor for IL-13, in keloid fibroblasts compared with fibroblasts from normal dermis. Ectopic expression of IL-13RA2 in keloid fibroblasts resulted in inhibition of STAT6 phosphorylation, cell proliferation, migration, invasion, extracellular matrix secretion, and myofibroblast marker expression, as well as an increase in apoptosis. Consistently, knockdown of IL-13RA2 in normal fibroblasts induced a keloidal status. Furthermore, both in vitro application and intratumoral injection of p-STAT6 inhibitor AS1517499 in a patient-derived xenograft keloid-implantation mouse model resulted in proliferation inhibition and tissue necrosis, apoptosis, and myofibroblast marker reduction. Collectively, this study elucidates the key role of IL-13RA2 in keloid pathology and inspires further translational research of keloid treatment concerning JAK/STAT6 inhibition.

## Graphical abstract

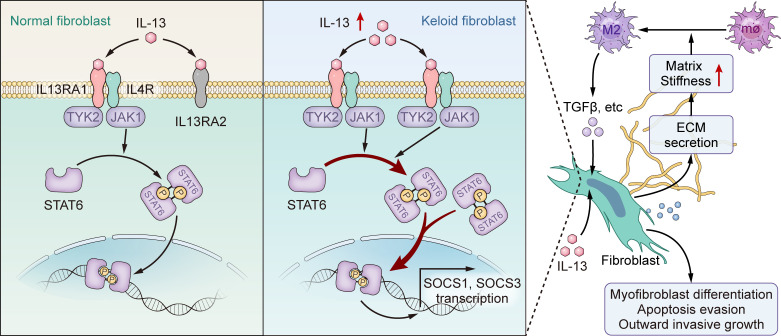

## Introduction

Keloids occur following reticular dermis injury and grow outward beyond the original wound, with features comparable to tumors; they are therefore referred as an over-healing fibroproliferative disease (1). Although suboptimal treatments (surgery combined with 5-fluorouracil chemotherapy or triamcinolone injection) can delay disease progression, the recurrence rate of keloids is still high, and patients suffer from long-term symptoms like itching and pain. Uncontrolled keloids could lead to functional impairments, which bring a heavy burden to life. To date, there are still no fully curative treatments for keloids (2).

Integrated wound healing is regulated by numerous transcription factors, cytokines, and growth factors (3). Fibroblasts exhibit distinct regulatory activities between keloid pathogenesis and normal wound healing (4–6). During wounding, dermal fibroblasts are activated to become myofibroblasts that express α-smooth muscle actin (α-SMA), proliferate and migrate to the wound, deposit complex extracellular matrices (ECMs), and create signaling niches (4, 7, 8). Once tissue integrity is reestablished, fibroblasts deactivate or initiate self-clearance by apoptosis (9). In contrast, keloid fibroblasts (KFs) remain activated, continuously secreting ECM and evading apoptosis by conducting prosurvival signals from the fibrotic microenvironment, resulting in an excess of scar tissue (10). Therefore, identifying signaling pathways underlying fibrogenesis of KFs may shed light on novel therapeutic strategies.

IL-13, a key driver of progressive fibrosis (11–13), has been found to be elevated in keloid patients (14). IL-13 has also been reported to induce production of antiapoptotic proteins and directly enhance myofibroblast function. In addition, IL-13 can induce the development of restorative macrophages (M2) through JAK/STAT6 signaling and M2 macrophages in turn facilitate the recruitment of IL-13–producing leukocytes to the wound (11, 13, 15, 16). Moreover, M2 macrophages have been reported to promote fibrosis through multiple ways, including TGF-β, matrix metalloproteinase (MMP), etc. JAK/STAT6 signaling is activated upon binding of IL-13 with its receptors IL-4R and IL-13RA1. Interestingly, IL-13RA2, a decoy receptor of IL-13 that lacks intracellular domains, has significantly higher affinity for IL-13 compared with the true receptors, IL-4R and IL-13RA1, and can impair downstream JAK/STAT6 signaling. When IL-13RA2 expression is low or absent, IL-13–dependent fibrosis is exacerbated (11, 15, 17). Diaz et al. reported that keloid lesions responded to dupilumab (IL-4/IL-13R antagonist) therapy (18); thereafter, a series of clinical trials of dupilumab in keloid treatments has been reported. However, the cellular and molecular mechanisms are not well studied.

In the present study, we have found that IL-13 signaling and the downstream JAK/STAT6 pathway are activated by the downregulation of IL-13RA2 in keloids. Inhibiting the phosphorylation of STAT6 and ectopic overexpression of IL-13RA2 alleviate keloid fibrogenesis by inducing apoptosis and suppressing the migration, invasion, and collagen synthesis of myofibroblasts in vitro and in vivo. These findings are consistent with a recent preclinical trail of JAK inhibitors to treat keloids and refine the key role of IL-13RA2 and JAK/STAT6 in keloid fibrogenesis, which may inspire further translational research of keloid treatment and wound healing.

## Results

### M2-like macrophages were enriched in keloids.

**Figure 1:**
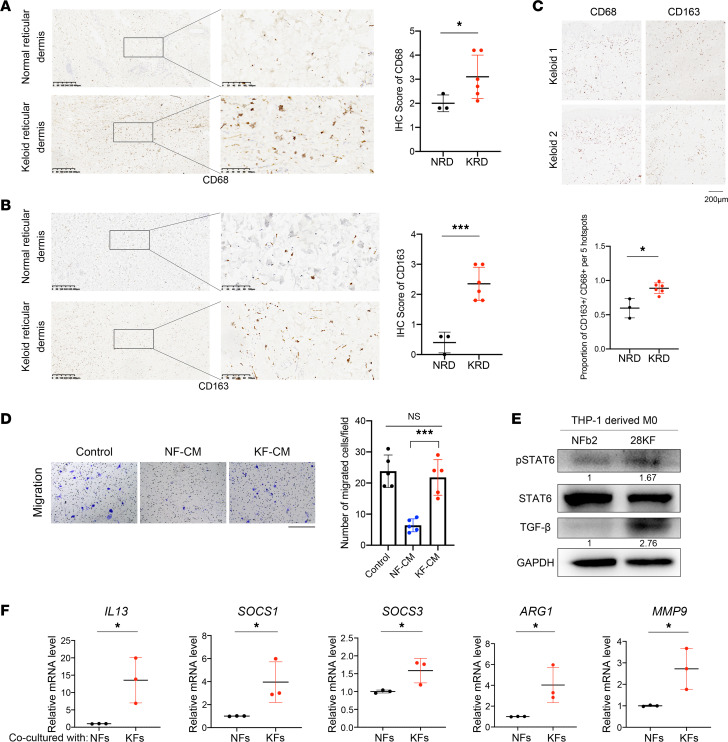
M2-like macrophages were enriched in keloids. (**A**) Representative images of IHC staining of CD68 (macrophages) in normal skin and keloid. Scale bars: 200 μm. Quantification by IHC scores shown on right. (**B**) Representative images of IHC staining of CD163 (M2-like macrophages) in normal skin and keloid. Scale bar: 200 μm. Quantification by IHC scores shown on right. (**C**) Representative images of IHC staining of CD68 and CD163 in the same samples. Scale bar: 50 μm. The ratio of CD163^+^/CD68^+^ representing percentage of M2-like/total macrophages was quantified by positive cell number/nuclei number (blue) per 5 hotspots and is shown below. (**D**) Transwell migration assay of PMA-induced THP-1 (M0) cells upon stimulation with NF-CM and KF-CM. Scale bar: 400 μm. Quantification of chemoattracted cells shown on right. (**E**) Expression of p-STAT6, STAT6, and TGF-β in THP-1–derived M0 cells when cocultured with indicated fibroblasts. Relative gray scale of p-STAT6/STAT6 and TGF-β/GAPDH are shown below the blots. (**F**) The mRNA levels of indicated genes in THP-1–derived M0 cells in different coculture systems (*n* = 3). *P < 0.05; ***P < 0.001 by 2-tailed Student’s *t* test. NS, not significant (*P* > 0.05).

Masson’s trichrome staining was conducted, and densely packed keloidal collagen bundles were found twisted and accumulated with ECM in keloids (Supplemental Figure 1, red arrows; supplemental material available online with this article; https://doi.org/10.1172/jci.insight.157091DS1). Consistent with a report by Li et al. (19), immunohistochemistry (IHC) revealed that both total macrophages (CD68^+^) and M2-like macrophages (CD163^+^) were enriched significantly in keloids, with M2-like macrophages being enriched more significantly (*P* < 0.001). Moreover, the CD163^+^/CD68^+^ ratio was higher in keloids compared with normal skins (*P* < 0.05), indicating that enriched macrophages in keloids were mostly the M2 type (Figure 1, A–C).

Primary fibroblasts from keloid (KFs) and normal skin dermis (NFs) were isolated, cultured, and conditioned medium was collected from NFs and KFs and ultrafiltrated using 3 kDa ultrafiltration tube, herein referred as NF-CM and KF-CM, respectively. Transwell assays revealed that only a few M0 macrophages (PMA-induced THP-1 cells) migrated upon NF-CM stimulation, while the number of chemoattracted M0 cells increased significantly upon KF-CM stimulation (*P* < 0.0001), similar to the positive control (DMEM with 10% FBS) (Figure 1D). These findings suggest that KFs can recruit macrophages, at least in part accounting for the enrichment of macrophages in keloids. The crosstalk between fibroblasts and macrophages has been reported to modulate the outcome of cutaneous wound healing. Thus, we constructed a coculture system containing THP-1–derived M0 cells and fibroblasts (NF or KF) and found the expression of p-STAT6 and TGF-β in M0 cells was upregulated when cocultured with KFs .TGF-β was regarded as the key regulator of keloid fibrogenesis (Figure 1E). Moreover, the mRNA levels of *IL13*, JAK/STAT6 target genes (*SOCS1* and *SOCS3*), and M2-like macrophage–related genes (*ARG1* and *MMP9*) were also upregulated significantly in THP-1–derived M0 cells when cocultured with KFs (Figure 1F). Furthermore, the IL-13 concentration was higher in the medium of the KF–M0 cell coculture, although it was not significant (Supplemental Figure 2). These results suggest that KFs may promote the differentiation of macrophages toward the M2-like subtype through STAT6 signaling. The increased expression of TGF-β may mediate the differentiation of macrophages toward the M2-like subtype and promote fibrosis, which needs further investigation.

### JAK/STAT6 signaling was activated in KFs.

**Figure 2:**
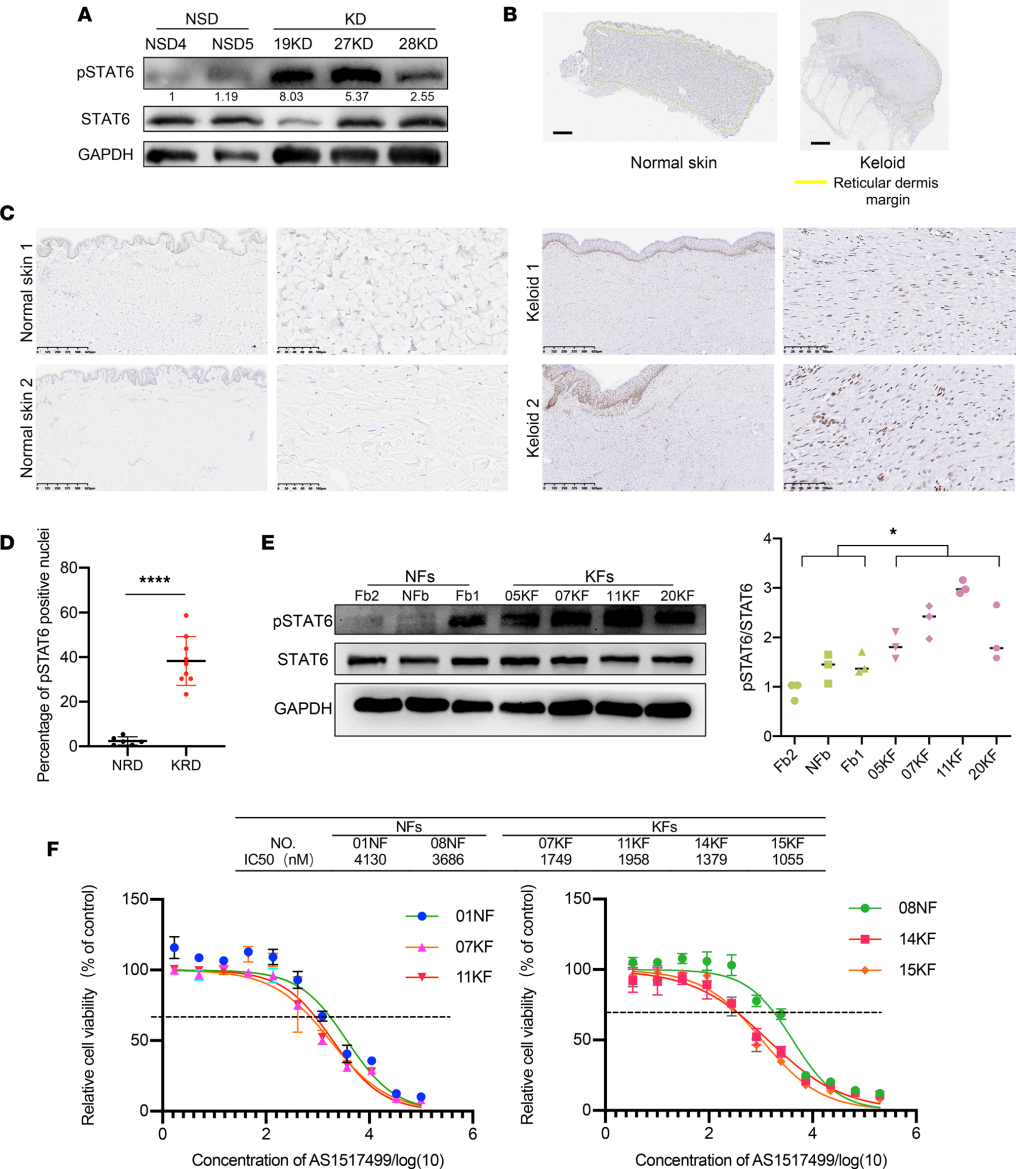
JAK/STAT6 signaling was activated in KFs. (**A**) Expression of p-STAT6 and STAT6 in normal skin dermis (NSD) and keloid dermis (KD) (*n* = 2 and 3, respectively). (**B**) HALO analysis of reticular dermis (circled in yellow lines) of normal skins (*n* = 6) and keloids (*n* = 9). Quantification of percentage of p-STAT6^+^ nuclei shown on right. (**C** and **D**) Representative images and quantification of p-STAT6 IHC staining of normal skins and keloids. Scale bars: 100 μm. (**E**) Expression of p-STAT6 and STAT6 in NFs (*n* = 3) and KFs (*n* = 4). Quantification of p-STAT6/STAT6 by relative gray value shown below. (**F**) Sensitivity of NFs and KFs to p-STAT6 inhibitor AS1517499. Cell proliferation was detected by CCK8 assay (*n* = 2 and 4, respectively). Error bars represent SD. Experiments were performed 3 times and in triplicate each time. *P < 0.05; ****P < 0.0001 by 2-tailed Student’s *t* test.

JAK/STAT6 signaling activation mediated by IL-4 and IL-13 is closely associated with M2 activation and polarization, and plays important roles in fibroproliferative disorders (15). We therefore examined the level of p-STAT6 in keloid tissues and healthy skins. Tissue protein lysates from both keloids and normal dermis were assayed by Western blotting, and p-STAT6 was found to be upregulated in keloids (Figure 2A). IHC staining of p-STAT6 also showed a significantly elevated expression in keloid reticular dermis (Figure 2, B–D). Similarly, the p-STAT6/STAT6 ratio was higher in KFs compared with NFs (Figure 2E). These findings suggest that JAK/STAT6 signaling may also be involved in keloid progression.

AS1517499, a specific inhibitor of p-STAT6, was used to treat primary fibroblasts at concentrations ranging from 3.387 nM to 200 μM for 3 days to analyze the sensitivity of primary fibroblasts to p-STAT6 inhibition. As shown in Figure 2F, KFs were more sensitive to AS1517499 compared with NFs, with IC_50_ values of 1055–1958 nM and 3688–4130 nM, respectively, indicating that KFs are more reliant on JAK/STAT6 signaling.

### IL-13RA2 was downregulated in KFs and negatively correlated with JAK/STAT6 activation.

**Figure 3:**
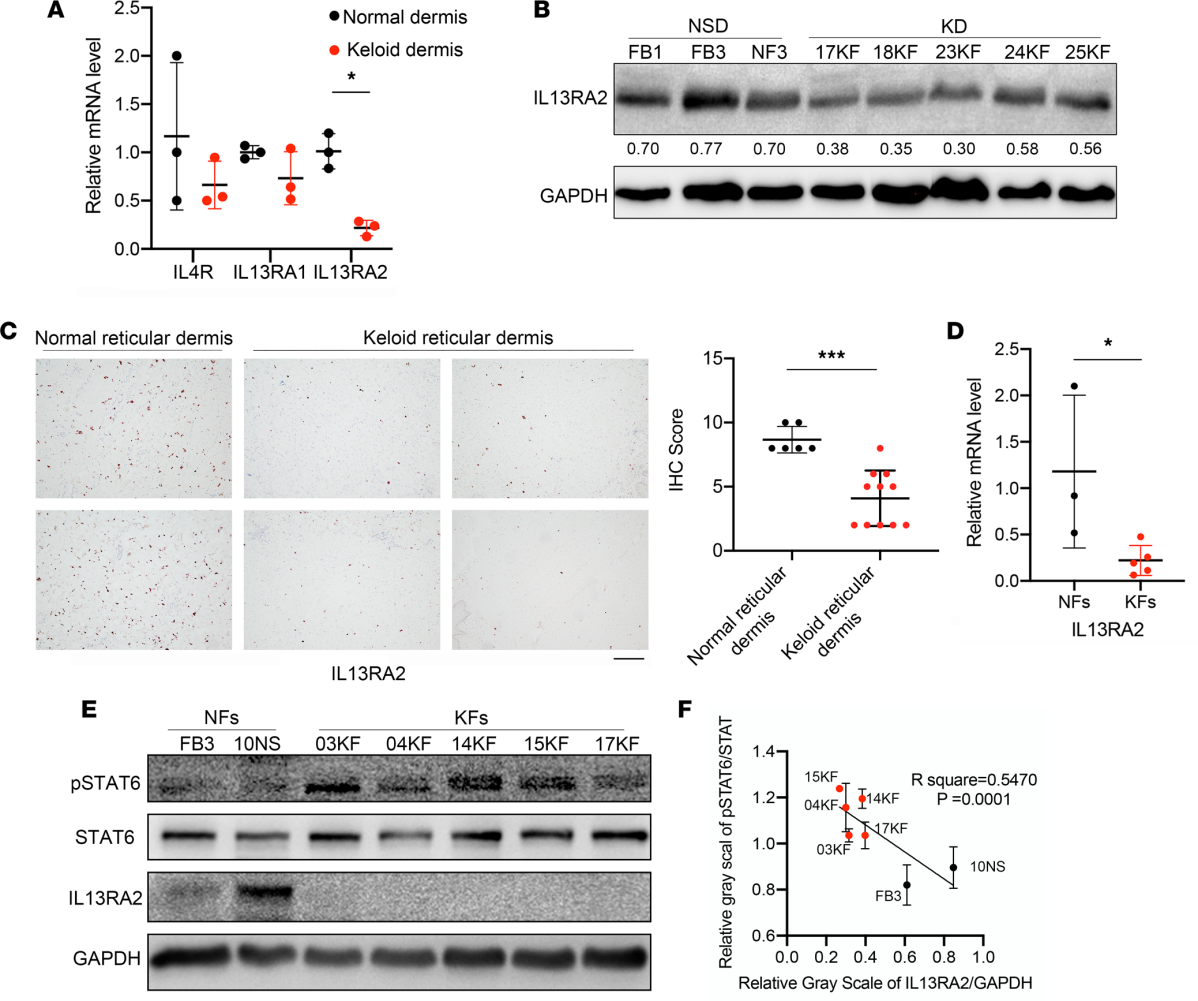
IL-13RA2 was downregulated in KFs and negatively correlated with p-STAT6. (**A**) The mRNA levels of *IL13RA2*, *IL13RA1*, and *IL4R* in normal skin dermis and keloids (*n* = 3 and 5, respectively). (**B**) The protein level of IL-13RA2 in normal skin dermis (NSD) and keloid dermis (KD). Quantification of gray scale of IL-13RA2/GAPDH is shown below. (**C**) Representative images of IL-13RA2 expression by IHC staining in normal skins and keloids. Scale bar: 50 μm. IHC scores of IL-13RA2 in normal skin (*n* = 6) and keloids (*n* = 11) is shown on right. (**D**) The mRNA level of *IL13RA2* in NFs and KFs (*n* = 3 and 5, respectively). (**E**) The protein level of IL-13RA2 in NFs (*n* = 3) and KFs (*n* = 4). (**F**) The negative correlation between IL-13RA2 and p-STAT6. Pearson’s correlation analysis of relative gray values is shown. Error bars represent SD. Experiments were performed 3 times. *P < 0.05; ***P < 0.001 by 2-tailed Student’s *t* test.

JAK/STAT6 signaling was activated upon binding of IL-4 and IL-13 with specific receptors. To explore the mechanism behind p-STAT6 upregulation, qRT-PCR was used to detect the mRNA levels of the IL-4/IL-13 axis, including *IL4*, *IL13*, *IL4R*, *IL13RA1*, and *IL13RA2* in keloid tissues and KFs. Neither *IL4* nor *IL13* mRNA was detectable in NFs or KFs (Ct value >35). However, *IL13RA2* mRNA was significantly lower in keloids than normal skins. Accordingly, quantitative analysis using gray values revealed that IL-13RA2 protein was downregulated in keloids compared with normal dermis (*P* < 0.05) (Figure 3, A and B). Moreover, IHC staining of keloids and normal dermis also showed that IL-13RA2 protein was decreased in keloids (Figure 3C).

In accordance, the *IL13RA2* mRNA level was also lower in KFs than NFs (*P* < 0.05) (Figure 3D). Furthermore, Western blot assay of NFs and NFs with Pearson’s correlation test of gray values revealed a negative correlation between IL-13RA2 expression and the p-STAT6/STAT6 ratio (Figure 3, E and F), suggesting an important role of IL-13RA2 in JAK/STAT6 activation in keloids.

### Overexpression of IL-13RA2 alleviated KF fibrosis-related cell behaviors.

**Figure 4:**
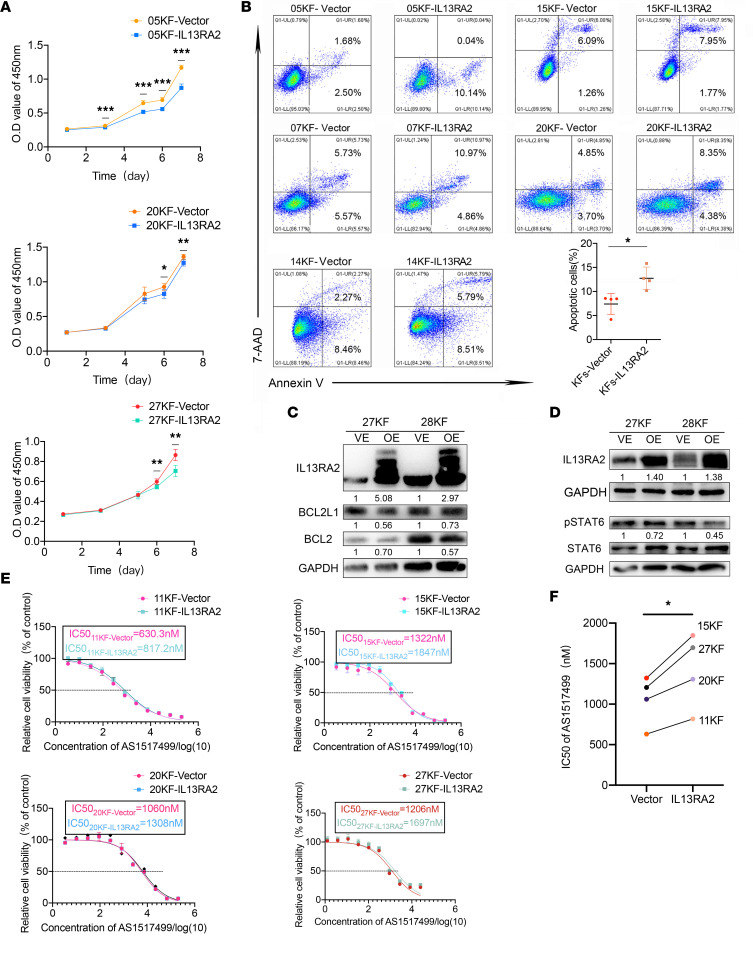
Overexpression of IL-13RA2 induced KF apoptosis. (**A**) Growth curves of KFs upon IL-13RA2 overexpression. (**B**) Annexin V/7-AAD double staining assay of KF-vector and KF-IL13RA2 cells (*n* = 5). Quantification of Annexin V–positive rates, representing apoptotic rates, is shown below. (**C** and **D**) Expression of p-STAT6, BCL2L1, and BCL2 in KFs upon IL-13RA2 overexpression. Quantifications of relative gray scale is shown below the blots. (**E** and **F**) IC_50_ values of AS1517499 in KF-vector and KF-IL13RA2 cells. *P < 0.05; **P < 0.01; ***P < 0.001 by paired, 2-tailed Student’s *t* test.

To investigate the function of IL-13RA2 in keloid pathogenesis, *IL13RA2* was overexpressed in KFs (KF-IL13RA2) from different patients using lentivirus. As shown in Figure 4A, *IL13RA2* overexpression suppressed KF proliferation. No significant difference was found in cell cycle progression between KF-IL13RA2 and KF-vector (Supplemental Figure 3). However, *IL13RA2* overexpression increased the apoptotic rate of KFs, as revealed by Annexin V/7-AAD staining (7.395% ± 1.857% in KF-vector vs. 12.74% ± 2.023% in KF-IL13RA2, *P* < 0.05), indicating that proliferation inhibition was due to increased apoptosis. In addition, Western blot assay also showed that *IL13RA2* overexpression decreased the expression of antiapoptotic proteins BCL2 and BCL2L1 (Figure 4, B and C). Importantly, p-STAT6 expression was decreased in KF-IL13RA2, demonstrating the role of IL-13RA2 in modulating p-STAT6 levels (Figure 4D). In addition, KF-IL13RA2 were less sensitive to AS1517499 but still more sensitive than NFs, as reflected by their IC_50_ values (Figure 4, E and F).

**Figure 5:**
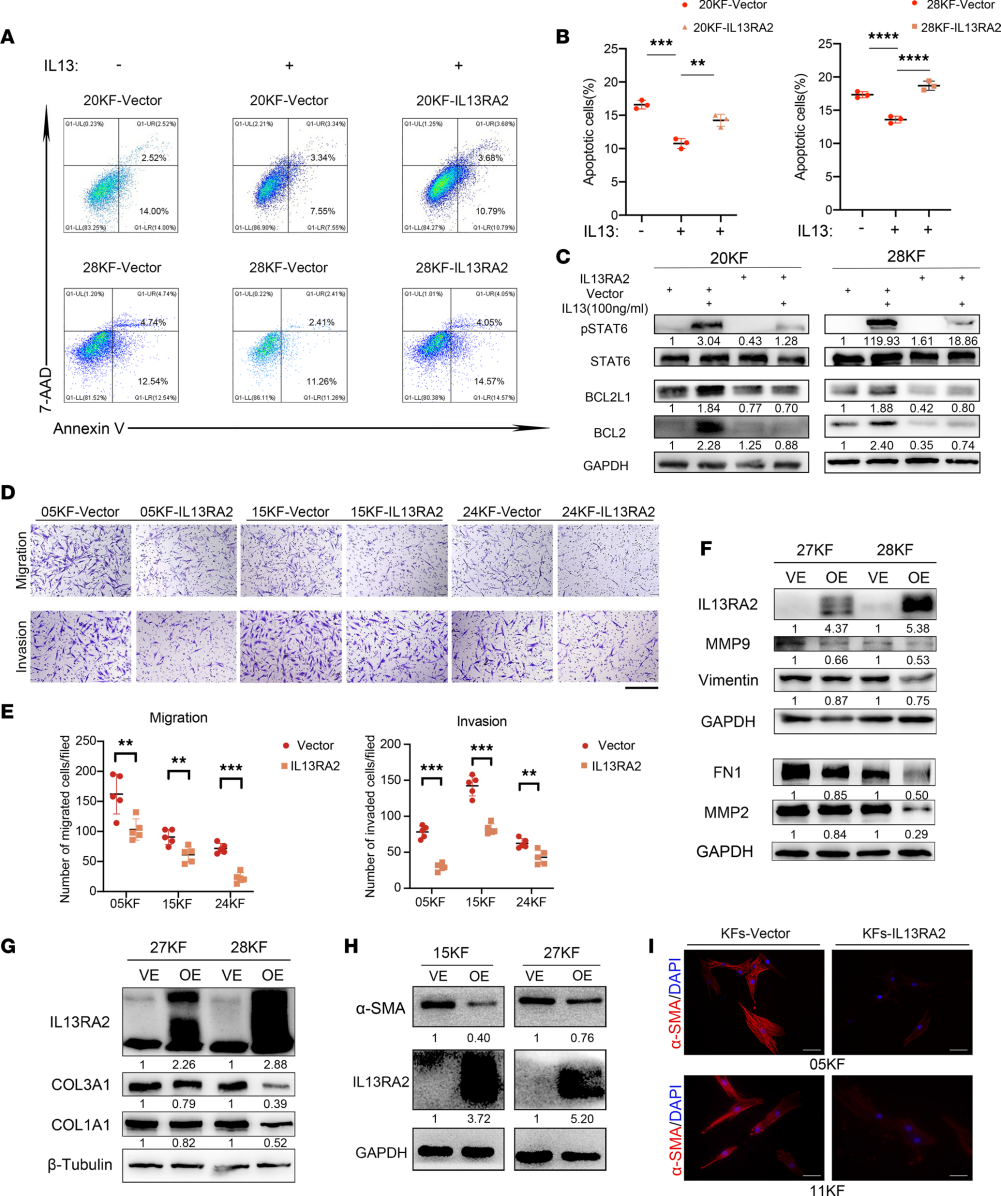
Overexpression of IL-13RA2 alleviated KF IL-13–related profibrotic cell behavior. (**A**) Annexin V/7-AAD double staining assay of KFs with or without IL-13 stimulation. (**B**) Quantification of Annexin V–positive rates, representing apoptotic rates. (**C**) Expression of p-STAT6, BCL2L1, and BCL2 in 20KF-vector and 20KF-IL13RA2 cells with or without IL-13 stimulation. (**D** and **E**) Migration and invasion of KF-vector and KF-IL13RA2 cells. Scale bar: 50 μm. Quantification of migrated and invaded cells is shown in **E**. (**F** and **G**) Expression of MMPs, COL3A1 and COL1A1, and mesenchymal markers FN1 and vimentin in KF-vector and KF-IL13RA2 cells. (**H**) Expression of α-SMA in KF-vector and KF-IL13RA2 cells. (**I**) Representative images of α-SMA immunofluorescent staining in KF-vector and KF-IL13RA2 cells. Scale bars: 100 μm. Error bars represent SD. Experiments were performed 3 times and at least in triplicate each time. **P < 0.01; ***P < 0.001; ****P < 0.0001 by 2-tailed Student’s *t* test.

We observed in the apoptosis assay that IL-13 stimulation markedly reduced spontaneous apoptosis of KF-vector, while overexpression of IL-13RA2 reversed this effect (Figure 5, A and B). The level of p-STAT6 expression was clearly upregulated upon IL-13 stimulation in KF-vector, but only slightly increased in KF-IL13RA2 (Figure 5C). In addition, BCL2L1 and BCL2 were upregulated upon IL-13 stimulation in KF-vector but not in KF-IL13RA2, consistent with the results observed in the apoptosis assay (Figure 5C). These data verify that IL-13RA2 negatively regulates JAK/STAT6 activation in KFs. Moreover, Transwell assays revealed that IL-13RA2 overexpression mitigated KF migration and invasion (Figure 5, D and E). Western blot assay also showed that IL-13RA2 overexpression reduced the expression of mesenchymal markers (FN1, vimentin, and α-SMA), MMPs (MMP2 and MMP9), and collagen markers (COL1A1 and COL3A1) in KFs (Figure 5, F and G). Furthermore, immunofluorescence and Western blot assay revealed that α-SMA expression (myofibroblast marker) was decreased in KF-IL13RA2 compared with KF-vector (Figure 5, H and I). These data suggest that IL-13RA2 alleviates fibrosis of keloids.

### Knockdown of IL13RA2 in NFs induced keloid-characteristic behaviors via JAK/STAT6 signaling.

**Figure 6:**
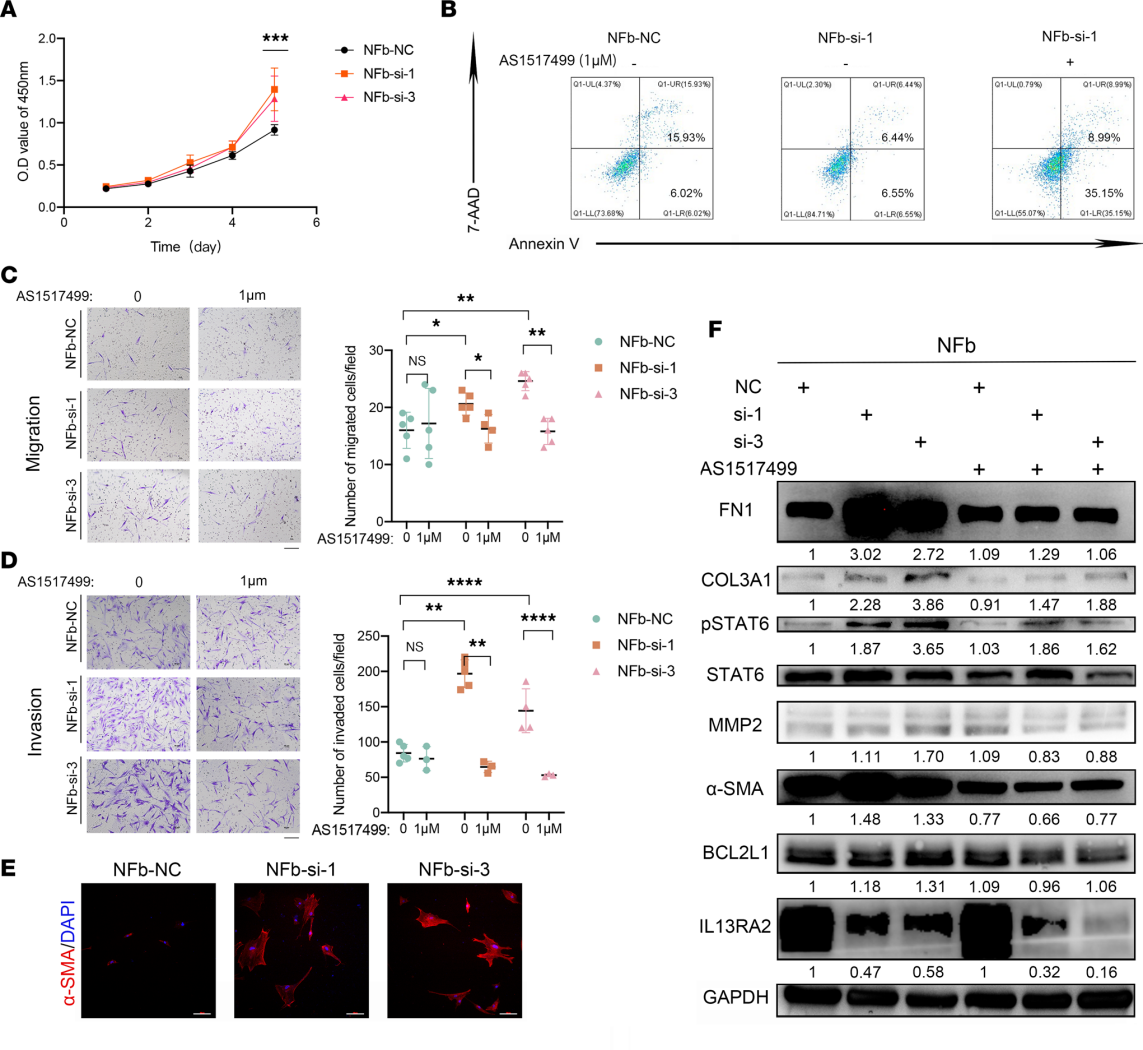
Knockdown of IL-13RA2 in NFs induced keloid-characteristic behaviors via JAK/STAT6. (**A**) Growth curves of NFb cells upon IL-13RA2 knockdown by CCK8 assay. NC, non-treatment control. (**B**) Annexin V/7-AAD double staining assay of NFb cells upon IL-13RA2 knockdown with or without AS1517499 treatment. NFb cells were treated with or without 1 μM AS1517499 for 16 hours before Annexin V/7-AAD double staining. (**C** and **D**) Migration and invasion of NFb upon IL-13RA2 knockdown with or without AS1517499 treatment. Cells (3 × 10^4^) were seeded in upper Transwell chambers and treated with or without 1 μM AS1517499 for 16 hours for the migration assay, while 6 × 10^4^ cells were seeded and treated with or without 1 μM AS1517499 for 22 hours for the invasion assay. Quantification of migrated and invaded cells is shown on the right. Scale bars: 100 μm. (**E**) Representative images of α-SMA immunofluorescent staining in NFb cells upon IL-13RA2 knockdown. Scale bars: 100 μm. (**F**) Western blot results of NFb cells upon IL-13RA2 knockdown. NFb cells were treated with or without 1 μM AS1517499 for 16 hours and then analyzed by Western blotting. Relative gray scales of p-STAT6/STAT6 and indicated protein/GAPDH are shown below the blots. Error bars represent SD. Experiments were performed 3 times and in triplicate each time. *P < 0.05; **P < 0.01; ***P < 0.001; ****P < 0.0001 by 2-tailed Student’s *t* test. NS, not significant (*P* > 0.05).

To comprehensively study the role of IL-13RA2 in keloid pathogenesis, we also assessed the effect of *IL13RA2* knockdown on the NF cell line NFb with siRNA (siRNA-IL13RA2). Both mRNA and protein levels of IL-13RA2 decreased after siRNA-IL13RA2 transfection (data not shown). As shown in Figure 6, A and B, knockdown of *IL13RA2* inhibited NFb apoptosis while it significantly promoted cell proliferation (*P* < 0.005). In addition, Transwell assays showed increased cell migration and invasion after knockdown of *IL13RA2* (Figure 6, C and D). Furthermore, Western blot assay confirmed that knockdown of *IL13RA2* upregulated the expression of BCL2L1 and BCL2, mesenchymal markers (FN1 and α-SMA), as well as COL3A1 (Supplemental Figure 4). Meanwhile, immunofluorescent staining confirmed that α-SMA expression was upregulated upon *IL13RA2* knockdown (Figure 6E).

As shown in Figure 6F, *IL13RA2* knockdown increased the expression of p-STAT6. To confirm whether IL-13RA2 functioned through regulation of JAK/STAT6 signaling, the p-STAT6 inhibitor AS1517499 was used to treat NFb cells with *IL13RA2* knockdown. As shown in Figure 6, B–D and F, AS1517499 treatment induced apoptosis and inhibited migration and invasion, while it downregulated the expression of FN1, MMP2, α-SMA, BCL2L1, and COL3A1 of NFb cells with *IL13RA2* knockdown. These data suggest that IL-13RA2 downregulation accelerates IL-13–induced fibrosis via JAK/STAT6 signaling.

### Inactivation of p-STAT6 with AS1517499 inhibited profibrotic activities of KFs.

**Figure 7:**
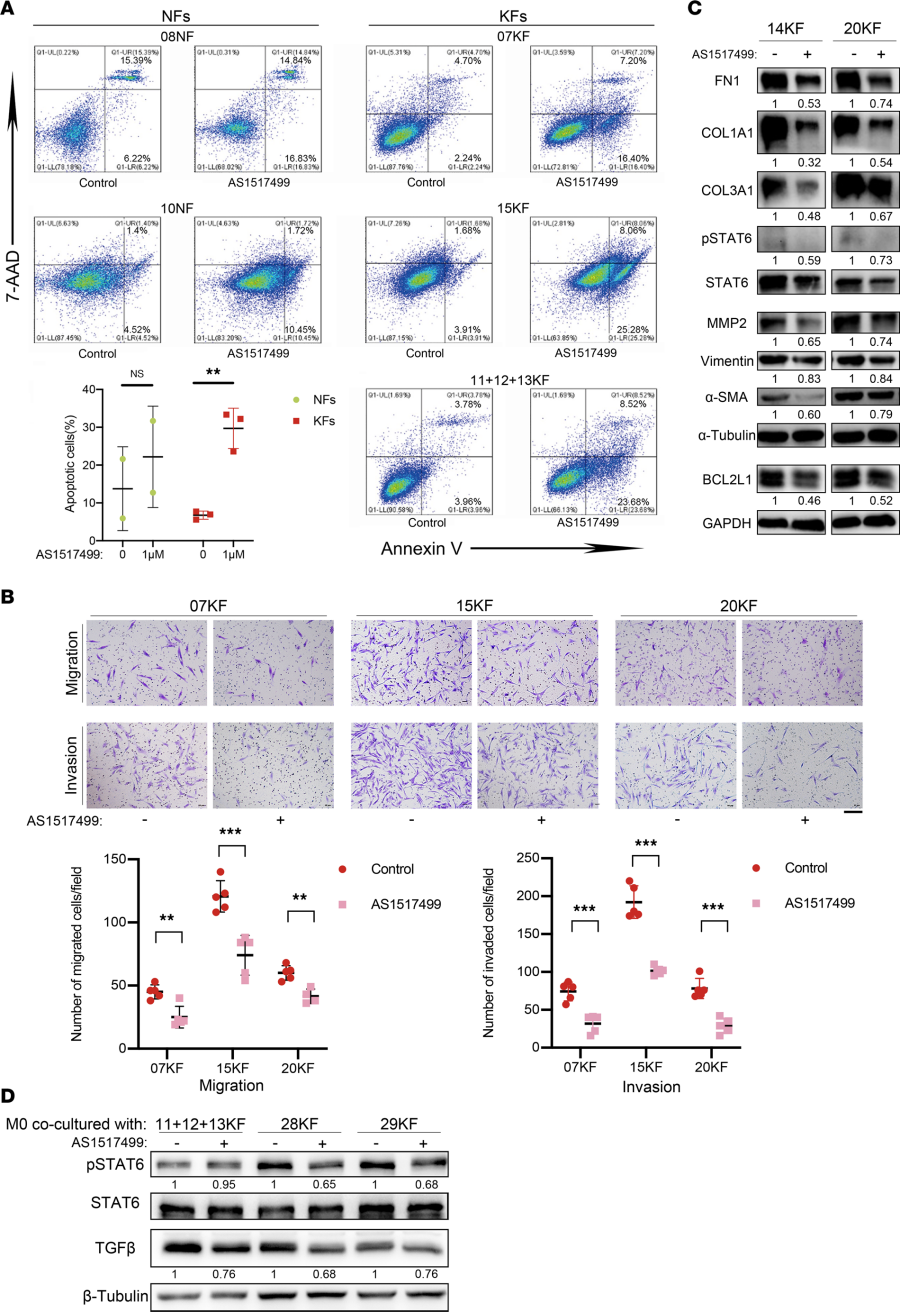
Inactivation of p-STAT6 with AS1517499 inhibited profibrotic activities. (**A**) Annexin V/7-AAD double staining assay of NFs and KFs treated with or without AS1517499. NFs and KFs were treated with or without 1 μM AS1517499 for 16 hours and then subjected to Annexin V/7-AAD staining. Quantification of Annexin V positivity, representing apoptotic rates, is shown below. (**B**) Migration and invasion of KFs with or without AS1517499 treatment. KFs were treated with or without 500 nM AS1517499 for 24 hours and then analyzed by Transwell assay. Scale bar: 50 μm. Quantification of migrated and invaded cells is shown below. (**C**) Expression of mesenchymal markers (FN1, vimentin), collagen (COL3A1, COL1A1), MMP2, BCL2L1, α-SMA, p-STAT6, and STAT6 in KFs upon AS1517499 treatment. KFs were treated with or without 500 nM AS1517499 for 24 hours and then analyzed by Western blotting assay. (**D**) Expression of p-STAT6, STAT6, and TGF-β in THP-1 cells when cocultured with pretreated KFs (800 nM AS1517499 for 48 hours or negative control). Relative gray scales of p-STAT6/STAT6 and indicated protein/GAPDH are shown below the blots. Error bars represent SD. Experiments were performed at least 3 times. **P < 0.01, ***P < 0.001 by 2-tailed Student’s *t* test. NS, not significant (*P* > 0.05).

To determine the effect of p-STAT6 on KFs’ profibrotic activities, p-STAT6 inhibitor AS1517499 was used in vitro. p-STAT6 expression in KFs was drastically downregulated by AS1517499 at 400 and 800 nM (Supplemental Figure 5). To fully inhibit JAK/STAT6 signaling, AS1517499 at 500 nM and 1 μM was used in the following experiments. As shown in Figure 7A, AS1517499 treatment (1 μM for 16 hours) significantly increased KF apoptosis (6.76% ± 1.09% in control vs. 29.71% ± 5.32% with AS1517499 treatment; *P* < 0.01), whereas the apoptosis of NFs was not significantly influenced (*P* = 0.1344). Moreover, AS1517499 treatment also significantly inhibited KF migration and invasion (Figure 7B). Western blot assay revealed that AS1517499 treatment in KFs led to downregulation of antiapoptosis protein BCL2L1, collagen (COL3A1 and COL1A1), mesenchymal markers (FN1, vimentin, and α-SMA), and matrix metalloproteinases (MMP2 and MMP9) (Figure 7C), indicating that AS1517499 treatment can alleviate collagen hyperproduction, promote myofibroblast deactivation, and inhibit profibrotic activities of KFs. Additionally, we also tested THP-1–derived M0 cells cocultured with KFs that were pretreated with AS1517499 or DMSO control, and found that p-STAT6 inactivation in KFs downregulated the expression of p-STAT6 and TGF-β in M0 cells (Figure 7D). Furthermore, the concentration of IL-13 was significantly downregulated in the coculture system with AS1517499-pretreated KFs (Supplemental Figure 6).

### AS1517499 inhibited fibrosis in keloid implantation model.

**Figure 8:**
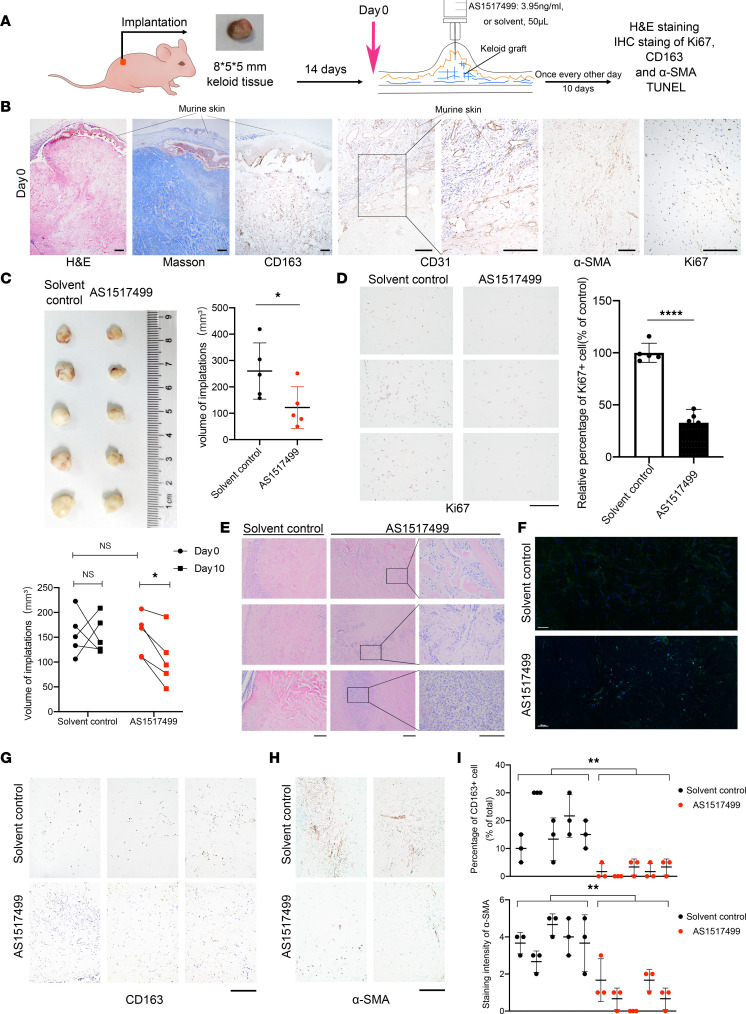
AS1517499 inhibited fibrosis in keloid implantation model. (**A**) Schematic diagram of animal experiment. (**B**) Representative images of H&E, Masson’s trichrome, and IHC staining for CD163, CD31, α-SMA, and Ki67 on day 0. Scale bars: 50 μm. (**C**) Images of transplantations after 10-day treatments. Quantification of volumes of xenografts in AS1517499-treated and control group is shown on the right (after dissection) and below (in vivo). (**D**) Representative images of IHC staining of Ki67 in AS1517499-treated and control group. Quantification of the percentage of Ki67^+^ cells is shown on the right. Scale bar: 100 μm. (**E**) Representative images of H&E staining of xenograft tissues in AS1517499-treated and control group. Scale bars: 50 μm. (**F**) Representative images of whole-slide scan of TUNEL staining in AS1517499-treated and control group. Cell nuclei were stained with DAPI (blue), and TUNEL-positive nuclei are stained in green. Scale bars: 100 μm. (**G** and **H**) Representative IHC staining of CD163 and α-SMA in AS1517499-treated and control group. Scale bars: 50 μm. (**I**) Quantification of percentage of CD163^+^ and α-SMA staining intensity. Error bars represent SD. *P < 0.05; **P < 0.01; ****P < 0.0001 by 2-tailed Student’s *t* test. NS, not significant (*P* > 0.05).

We established a keloid implantation model in BALB/c nude mice using fresh keloid tissues to assess the effect of p-STAT6 inhibition on keloid progression in vivo (Figure 8A). Low expression of IL-13RA2 in patient-derived keloids was also verified by IHC (Supplemental Figure 7). Fourteen days after implantation, IHC staining showed that CD31^+^ lumen-like cells were found in the confluent area of human and murine skins, suggesting that vascular anastomosis formed between the keloid implantation and murine skins. Importantly, keloid implantations revealed uniform pathological characteristics similar to that of patient keloids confirmed by H&E, Masson’s trichrome staining, and CD163 IHC staining (Figure 8B). Moreover, several papillary dermis cells underwent apoptosis, while only a few reticular fibroblasts underwent apoptosis, in line with what is seen in patients with keloids (Supplemental Figure 8).

After 10 days of AS1517499 treatment, the volume of implantations reduced significantly (*P* < 0.05), while no significant change was observed in the control group (Figure 8C). In accordance, the percentage of Ki67^+^ dermal fibroblasts was reduced by AS1517499 treatment to 32.83% of the control group (*P* < 0.0001), indicating that suppression of p-STAT6 inhibited proliferation of KFs in vivo (Figure 8D). H&E staining showed higher immune cell aggregation in the AS1517499 group, consistent with an observed increase in local tissue necrosis (Figure 8E). In addition, TUNEL staining showed that AS1517499 induced more fibroblast apoptosis in the whole layer of keloid-implanted dermis (Figure 8F). Moreover, AS1517499 treatment inhibited M2 macrophages, with CD163 staining virtually negative (Figure 8, G and I). IHC staining of α-SMA showed that AS1517499 treatment reduced the intensity and positivity rate of α-SMA, indicating a potent antimyofibroblast effect of p-STAT6 inhibition with AS1517499 treatment (Figure 8, H and I).

## Discussion

The pathogenic mechanisms underlying keloid formation and growth are not fully elucidated. It has been suggested that chronic inflammation as well as persistence of activated fibroblasts drive over-healing processes, leading to fibrosis (4, 5). Inspired by the accumulation of profibrotic M2 macrophages in keloid dermis, we aimed to explore the possible roles of the accumulation of M2 macrophages and found that it was associated with KFs.

Consistent with previous findings (19), we found that in patient keloid biopsies, M2 macrophages predominated. KF-CM was more potent in chemoattracting PMA-induced THP-1 cells (M0 cells), indicating the pivotal role of KFs in abnormal macrophage infiltration, although the detailed mechanism remains unknown. KFs could trigger p-STAT6 activation of M0 cells with upregulation of M2-related genes, and importantly, TGF-β expression. M2 macrophages have been found to promote fibroblast proliferation and myofibroblast activation with elevated TGF-β secretion (20–23). Whether media from M2 macrophages are capable of stimulating KFs is worthy of further investigation. Moreover, Shook et al. (23) confirmed that proliferation and heterogeneity of myofibroblasts were supported by macrophages during skin repair. Therefore, we propose that KFs create a profibrotic microenvironment to attract circulatory monocytes and polarize monocyte-derived macrophages to the M2 type, which in turn supports their own abnormal activation and the invasive growth of KFs.

We found that JAK/STAT6 signaling, which is known to induce M2 polarization, was activated in keloid tissues and KFs. Moreover, we found that KFs relied more on JAK/STAT6 signaling to proliferate than NFs in vitro. Enhanced antiapoptotic ability and persistent activation of fibroblasts are important pathogenic mechanisms of keloid fibrosis (10, 24, 25). During skin repair, myofibroblasts derived from multiple precursors migrate, proliferate, and deposit ECM. In normal wound healing, myofibroblasts disappear once reepithelialization is complete; however, in keloids, myofibroblasts fail to quiesce or undergo apoptosis, which is regarded as a hallmark of fibrotic diseases (8, 9). We found that inhibition of STAT6 phosphorylation with AS1517499 effectively induced the apoptosis of KFs and decreased the level of the antiapoptotic protein BCL2L1. Furthermore, myofibroblast marker α-SMA was reduced, cell migration and invasion were inhibited, while the mesenchymal markers and MMPs were downregulated by AS1517499. These findings demonstrate that JAK/STAT6 contributes to activation, proliferation, and invasive migration of KFs. MMP2 and MMP9 have the ability to degrade the basement membrane; thus, we presume that the reduction of MMPs could be associated with inhibition of invasion.

JAK/STAT6 activation classically associates with the IL-4/IL-13 axis. Despite that the expression of IL-4/IL-13 receptors IL-4R and IL-13RA1 showed no difference between keloids and normal skins, we demonstrated that IL-13RA2 expression was decreased in keloids as well as primary cultured KFs. Furthermore, IL-13RA2 downregulation was negatively correlated with p-STAT6 expression, while IL-13RA2 overexpression in KFs led to p-STAT6 downregulation, proliferation inhibition, spontaneous apoptosis, reduced migration and invasion, collagen secretion alleviation, and α-SMA downregulation. Moreover, IL-13RA2 overexpression also impaired the prosurvival effect of IL-13. On the other hand, IL-13RA2 knockdown in NFs upregulated p-STAT6 and elicited keloid-like profibrotic cell behaviors, with significant upregulation of antiapoptotic proteins as well as FN1, MMP2/9, α-SMA, and COL3A1, which was reversed by p-STAT6 inhibition with AS1517499. These results demonstrate that IL-13RA2 downregulation is a key driver of keloid pathogenesis via activation of JAK/STAT6. Interestingly, p-STAT6 inhibition in KFs reduced the p-STAT6 expression of M0 cells. Consistent with our previous finding that KFs drove p-STAT6 activation of THP-1 cells, we speculated that a possible positive feedback loop exists between KFs and profibrotic M2-like macrophages in keloid fibrogenesis via the IL-13/STAT6 axis and the deficient IL-13RA2 expression of KFs, which warrants further investigation.

Heretofore, it remains controversial whether dupilumab is effective in treating keloids, and the specific mechanism is not known. Our study indicates that IL-13R antagonists along with JAK inhibitors could potentially modify keloid disease through inhibiting the IL-13/STAT6 axis of KFs and profibrotic M2 polarization. Importantly, our in vivo experiments with AS1517499 have confirmed its expected antifibrotic effects, including inhibition of keloid proliferation, induction of cell apoptosis, and decrease in α-SMA^+^ fibroblasts, highlighting the clinical and translational prospect of our findings.

It is also important to understand the fate of the missing α-SMA^+^ myofibroblasts upon p-STAT6 inhibition or ectopic expression of IL-13RA2. According to recent research (26), during wound healing, myofibroblasts become fat cells and reduce scar formation. We analyzed microarray data of 7 active keloid lesions versus adjacent normal skin tissues (GEO GSE90051) and found pathways related to fat biosynthesis, metabolism, and PPARG as top downregulated pathways by both GO and KEGG enrichment analyses (Supplemental Figure 9). Thus, we speculate that myofibroblast adipogenesis may be inhibited, at least in part, due to JAK/STAT6 activation in keloids, resulting in excess scar formation, which requires further investigation. Moreover, our in vitro study found that JAK/STAT6 activation promoted ECM production, with elevated expression of FN1, COL1A1, and COL3A1, all of which could increase ECM stiffness. Atcha et al. (27) reported that stiff substrates enhanced IL-4/IL-13–induced STAT6 activation. We thus speculate that increased stiffness coordinates with IL-13 in the microenvironment to drive M2 polarization in keloids. Finally, we chose AS1517499 to inhibit JAK/STAT6 in the patient-derived xenograft (PDX) model instead of directly regulating IL-13RA2 expression because there is still a lack of established animal models of keloids (28), and we believe organoid cultures and PDX will bridge current difficulties (29).

**Figure 9:**
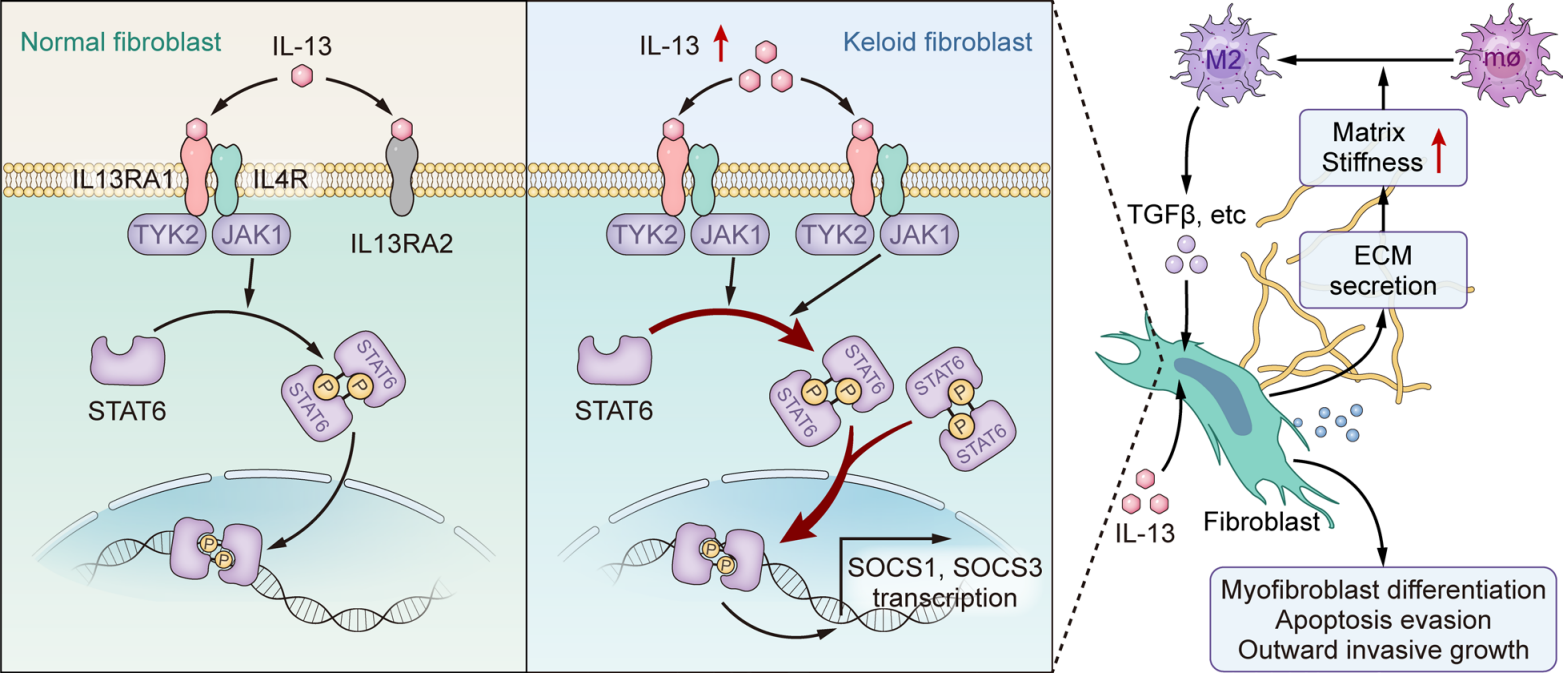
Schematic diagram of possible mechanism of IL-13RA2 in modulating JAK/STAT6 in keloids and its downstream effects. Downregulation of IL-13RA2 in fibroblasts aggravates fibrosis via JAK/STAT6. The increased matrix stiffness due to ECM secretion that is caused by JAK/STAT6 activation along with elevated IL-13 in conjunction leads to M2 polarization. M2 macrophages provide prosurvival and profibrotic cytokines, supporting keloidal cell behavior and further pathological fibrosis of keloids.

In conclusion, our study demonstrates that downregulation of IL-13RA2 in KFs results in abnormal profibrotic characteristics of KFs through activating JAK/STAT6 (Figure 9). Overall, the role of IL-13RA2 described here may enable a deeper understanding of the pathogenesis of keloids, which may in turn yield benefits for clinical transformation.

## Methods

### Clinical specimens.

All keloid and normal tissues were collected at the First Affiliated Hospital of Sun Yat-Sen University from 2020 to 2021.

### Reagent.

Reagents used in this study are listed in Supplemental Table 2.

### Primary fibroblast isolation and culture.

Primary KFs and NFs were obtained from fresh tissue specimens, with the patients’ clinical characteristics listed in Supplemental Table 1. Briefly, epidermis was removed with sterile scissors, and dermis was then cut thoroughly and incubated in DMEM supplemented with 0.2% collagenase I and 10% fetal bovine serum (FBS) at 37°C for 4 hours. Thereafter, dissolved cells were collected, filtered with a 70 μm cell strainer, centrifuged at 1000 rpm for 5 minutes, and suspended in DMEM with 10% FBS and cultured in a humidified atmosphere at 37°C with 5% CO_2_. Primary cells were used in subsequent experiments from passages 2 to 6.

### THP-1 cell culture, PMA stimulation, and coculture with fibroblasts.

THP-1 cells were purchased from ATCC, cultured in RPMI 1640 with 10% FBS and supplemented with 0.05 mM β-mercaptoethanol (β-ME). To generate M0 cells, 1 × 10^6^ THP-1 cells were suspended in medium with 150 ng/mL PMA (Sigma Aldrich, P8139) for 48 hours and then replaced with fresh medium without β-ME for 24 hours in 0.4 μm Transwell chambers (JET, TCS016006). Meanwhile, 2 × 10^5^ fibroblasts were seeded in 6-well plates, and after adhesion, pretreated with fresh medium with 800 nM AS1517499 or control for 48 hours. Both THP-1–derived M0 cells and fibroblasts were washed with PBS 3 times. Thereafter, the Transwell chambers containing M0 cells were carefully moved to the upper chamber of 6-well plates and 1.5 mL and 2.5 mL RPMI 1640 medium were added to the upper chambers and lower chambers, respectively. The cells were cocultured for 48 hours and then collected.

### RNA extraction and RT-PCR.

Fresh specimens were collected in RNALater (Thermo Fisher Scientific, AM7024) and stored at –80°C. Tissue RNA extraction was performed with a Tissue RNA Purification Kit Plus (EZBioscience, EZB-RN001-plus) according to the manufacturer’s instructions, and cell RNA extraction was performed with an Express RNA Purification Kit (EZBioscience, B0004DP). RNA concentration was quantified using Nanodrop 2000 (Thermo Fisher Scientific) and 1 μg RNA was used for reverse transcription using a Color Reverse Transcriptase Kit (EZBioscience, A0010CGQ). Gene expression was quantified by real-time PCR using SYBR Green qPCR Mix (EZBioscience, A0012-R2) as previously described. The primers used for the detected genes are listed in Supplemental Table 3.

### Protein extraction and immunoblotting.

Tissues were homogenized with a homogenizer. Proteins were extracted by incubation with RIPA lysis buffer (Beyotime Biotechnology, P0013B) supplemented with protease inhibitors (Roche, 05056489001) and phosphatase inhibitors (Roche, 04906837001) for 30 minutes on ice, followed by centrifugation at 14940*g* for 30 minutes. Supernatants were quantified using a BCA Protein Assay Kit (Thermo Fisher Scientific, 23227).

Immunoblotting was performed as previously described. Briefly, after boiling in loading buffer (Yeasen, 20315ES05) at 95°C for 10 minutes, the proteins were resolved by SDS-PAGE and then transferred onto PVDF membranes. Then, the membranes were blocked in TBST with 5% nonfat milk for 1 hour at room temperature before overnight incubation with primary antibodies at 4°C. Thereafter, the membranes were incubated with HRP-conjugated secondary antibodies at room temperature for 1 hour. The immune complexes were detected by ChemiDoc Touch Gel Imaging System (Bio-Rad, 1708370) and the relative protein expression was calculated through gray analysis using ImageJ software (NIH). See complete unedited blots in the supplemental material.

### CM collection.

Fibroblasts were seeded in 100 mm dishes and cultured to reach a confluence of 90%. Then, the cells were washed with PBS, and cultured for 24 hours in 10 mL FBS-free DMEM. Thereafter, the media were collected, centrifuged at 14940*g* for 10 minutes at 4°C, and then the supernatants were ultrafiltered using 3 kDa ultrafiltration tubes at 5055*g* until 10 mL medium was concentrated to 150 μL and stored at –80°C. KF-CM and NF-CM were collected and added to the lower chambers as stimulators in Transwell assays of PMA-induced THP-1 cells.

### Transfection and lentiviral infection.

Because primary fibroblasts were difficult to transfect with plasmids, overexpression of *IL13RA2* was achieved with lentivirus purchased from (GENECHEM, GIDL0219876). Briefly, 1.6 × 10^5^ fibroblasts were seeded in 6-well plates, and when the cells reached 50% confluence, 20 μL of 1 × 10^8^ TU/mL overexpression or control virus solutions were added with 4 μg/mL polybrene (Sigma-Aldrich, 107689). Twelve hours after infection, the media were replaced with fresh media. Knockdown of *IL13RA2* was achieved by siRNA against *IL13RA2* (Transheep, SI2108081). NFs were transfected with siRNA oligonucleotides (Supplemental Table 4) using INTERFERin (Polyplus, 409-10) according to the manufacturer’s instructions.

### CCK8 cell proliferation assay.

One thousand fibroblasts were seeded into each well of 96-well plates, and cell proliferation was measured by detection of OD450 with a BioTek Synergy H1 at indicated time points using the CCK8 (EZBioscience, EZB-CK8) assay according to the manufacturer’s instructions.

### Cell apoptosis assay.

Annexin V–APC/7-AAD double staining was performed to detect cell apoptosis. Briefly, cells were seeded in 6-well plates to adhere overnight, and then treated with AS1517499 or vehicle. The supernatants were collected, and cells were recovered via trypsinization without EDTA. Annexin V–APC/7-AAD Apoptosis Detection Kit (KeyGEN, KGA1023) was used according to the manufacturer’s instructions and cell apoptosis was measured by flow cytometry.

### Transwell migration and invasion assay.

Migration and invasion assays were conducted using cell culture inserts (Falcon, 35097) with and without Matrigel (Corning, 354480), according to the manufacturers’ protocol. Briefly, for migration assay, 200 μL of cell suspensions without FBS (for THP-1 cells, 1 × 10^5^ cells pretreated with 100 ng/mL PMA (Sigma-Aldrich, P8139) for 24 hours and rested in RPMI 1640 with 10% FBS for another 24 hours; for KFs and NFs, 3 × 10^4^ cells pretreated with 500 nM AS1517499 (or DMSO control) were added to the upper 24-well chambers and incubated for 16 hours. For invasion assay, 4 × 10^4^ KFs or 6 × 10^4^ NFs in 200 μL of cell suspension without FBS were added to the upper 24-well chambers and incubated for 23 hours. The migrated or invaded cells were then stained with 0.2% crystal violet, photographed, and counted under a microscope (NIKON ECLIPSE 80i).

### Immunofluorescence assay.

For immunofluorescence assays, cells were cultured on glass-bottom dishes to attach overnight, and then fixed with 4% paraformaldehyde. The cells were treated with 0.1% Triton X-100 for 10 minutes at room temperature and incubated with anti–α-SMA primary antibody (Abcam, ab124964; 1:500) in PBST with 5% BSA at 4°C overnight. The cells were subsequently stained with matched secondary antibody (goat anti-rabbit IgG H&L, Alexa Fluor 594, Abcam, ab150080; 1:1000), followed by DAPI (Thermo Fisher Scientific, 62247) staining. The slides were then visualized and photographed using a fluorescence imaging system (NIKON Eclipse Ni-U).

### Masson’s trichrome staining, IHC staining, and TUNEL staining.

Masson’s trichrome staining was conducted using a Masson kit (Servicebio, G1006). Tissue biopsies (thickness = 3 μm) were placed on standard microscopy slides and stained following the manufacturer’s protocols. IHC staining was performed as previously described (30). The sections (thickness = 3 μm) were incubated with rabbit anti–p-STAT6 antibody (Abcam, ab263947; 1:1000), rabbit anti–IL-13RA2 antibody (Cell Signaling Technology, 85677; 1:400), rabbit anti-CD68 antibody (Cell Signaling Technology, 76437; 1:800), rabbit anti-CD163 antibody (Cell Signaling Technology, 93498; 1:800), rabbit anti-CD31 antibody (Cell Signaling Technology, 77699; 1:800), rabbit anti-Ki67 antibody (Abcam, ab15580; 1:200), and rabbit anti–α-SMA (Abcam, ab124964; 1:1000). The slides were scanned at ×20 magnification using a Jiang Feng slide scanner. Scoring of IHC was focused on reticular dermis. The staining results were independently and blindly reviewed by 2 clinical pathologists without knowledge of clinical data and other results. If the results were inconsistent, the 2 physicians would review the staining slide and reach an agreement through discussion. Cytoplasmic or membranous staining intensity of CD68, CD163, IL-13RA2, and α-SMA was categorized as follows: negative as 0, weak as 1, moderate as 2, and strong as 3. The percentage of stained cells was categorized as follows: 0 for <5% staining, 1 for 5%–25% staining, 2 for 26%–50% staining, 3 for 51%–75% staining, and 4 for 76%–100% staining. The proportion and intensity scores were then multiplied to generate a total score. Five fields with the highest percentage of CD68 or CD163 staining of each slide were selected and scored, with CD163^+^ percentage/CD68^+^ percentage standing for the M2/total macrophage ratio. Reticular dermis of both normal skins and keloids were selected and digitally annotated using HALO image analysis software v.2.3 (Indica Labs). To quantify p-STAT6^+^ nuclei at single-cell resolution, a digital image approach based on the HALO multiplex IHC v.1.2 base algorithm was developed. Color deconvolution was performed both for the nuclear counterstain and the DAB product. The p-STAT6^+^ percentage was calculated as p-STAT6^+^ nuclei/total cells. TUNEL staining was conducted using a TUNEL staining kit (Elabscience, E-CK-A320) according to the manufacturer’s instructions. The slides (thickness = 3 μm) were scanned with a Vectra Polaris (Akoya) under ×20 magnification.

### Animal experiments.

Twelve BALB/c female mice at the age of 6–8 weeks were purchased from Guangdong Gempharmatech Co., Ltd and bred in specific pathogen–free conditions. Keloid specimens were freshly removed from the epidermis with sterile scissors and cut into uniform pieces (8 × 5 × 5 mm), with the rest collected as initial controls. Keloid pieces were then implanted into nude mice subcutaneously. Fourteen days after implantation, the longest and shortest diameter of the implantations were measured, and the volumes of the xenografts were calculated using the formula *V* = 0.52 × (length × diameter^2^). The mice were randomly divided into the experimental and the control groups with equal mean volumes of xenografts. Two mice were euthanized for model verification, with the day of grouping set as day 0. The mice of the experimental group were subcutaneously injected with 50 μL AS1517499 (3.95 mg/mL in 5% DMSO, 40% PEG-300, 5% Tween 80, and 50% ddH_2_O) every other day, while the mice of the control group were injected with solvent. On day 10, the experiment was terminated, and the xenografts were embedded in paraffin and subjected to H&E, Masson’s trichrome, and TUNEL staining, as well as IHC staining of CD31, Ki67, CD163, and α-SMA.

### Statistics.

All analyses were conducted using GraphPad Prism software, version 8.0, and all statistical tests were 2-sided, with *P* less than 0.05 considered statistically significant. The data are presented as mean ± SD. The IHC score was analyzed by Wilcoxon’s test and Pearson’s correlation analysis was used to calculate the correlation coefficient (*r*) and the *P* value between IL-13RA2 and p-STAT6 relative gray scores after Gaussian distribution was proven. The IC_50_ value of AS1517499 was estimated using the nonlinear regression fitting method. Student’s unpaired *t* test or multiple *t* test was used if not specified.

### Study approval.

The use of clinical materials, including primary KFs and NFs obtained for research purposes, was approved by the First Affiliated Hospital of Sun Yat-Sen University Ethics Committee No. 2019 [300]. All patients were provided written informed consent prior to participation in the study. All animal experiments were approved by the Institutional Animal Care and Use Committee (IACUC) of the First Affiliated Hospital of Sun Yat-Sen University No. 2019 [300].

### Data availability statement.

Data of this study will be shared upon publication.

## Author contributions

HC, LZ, PH, HL, and QT designed the study. HC, PH, JH, SX, RZ, HL, YZ, and QT collected patients’ tissues. HC and PH performed the in vivo experiment. HC, LZ, and HM performed histopathological analysis and pathological tests. HC and LZ performed the in vitro experiments with assistance from JH, RW, SX, RZ, and HL. HC, LZ, and PH verified the data of this study. RW and QT provided reagents. HL and QT analyzed and interpreted the data. HC and LS wrote the draft of the manuscript. QT and HL revised the manuscript. All authors have reviewed the manuscript and approved the final version.

## Supplementary Material

Supplemental data

## Version 1 02/09/2023

In-Press Preview

## Version 2 03/22/2023

Electronic publication

## References

[1] Ogawa R (2017). Keloid and hypertrophic scars are the result of chronic inflammation in the reticular dermis. Int J Mol Sci.

[2] Bijlard E (2015). Intralesional 5-fluorouracil in keloid treatment: a systematic review. Acta Derm Venereol.

[3] Gurtner GC (2008). Wound repair and regeneration. Nature.

[4] Buckley CD (2001). Fibroblasts regulate the switch from acute resolving to chronic persistent inflammation. Trends Immunol.

[5] Henderson NC (2020). Fibrosis: from mechanisms to medicines. Nature.

[6] Liu X (2022). Single-cell RNA-seq reveals lineage-specific regulatory changes of fibroblasts and vascular endothelial cells in keloids. J Invest Dermatol.

[7] Rodrigues M (2019). Wound healing: a cellular perspective. Physiol Rev.

[8] Shaw T, Martin P (2016). Wound repair: a showcase for cell plasticity and migration. Curr Opin Cell Biol.

[9] Hinz B, Lagares D (2020). Evasion of apoptosis by myofibroblasts: a hallmark of fibrotic diseases. Nat Rev Rheumatol.

[10] Feng Y (2020). Targeted apoptosis of myofibroblasts by elesclomol inhibits hypertrophic scar formation. EBioMedicine.

[11] Wynn TA, Ramalingam TR (2012). Mechanisms of fibrosis: therapeutic translation for fibrotic disease. Nat Med.

[12] Lloyd CM, Snelgrove RJ (2018). Type 2 immunity: expanding our view. Sci Immunol.

[13] Borthwick LA (2013). Cytokine mediated tissue fibrosis. Biochim Biophys Acta.

[14] Nangole FW (2021). Multiple cytokines elevated in patients with keloids: is it an indication of auto-inflammatory disease?. J Inflamm Res.

[15] Wynn TA (2004). Fibrotic disease and the T(H)1/T(H)2 paradigm. Nat Rev Immunol.

[16] Salmon-Ehr V (2000). Implication of interleukin-4 in wound healing. Lab Invest.

[17] Nguyen JK (2020). The IL-4/IL-13 axis in skin fibrosis and scarring: mechanistic concepts and therapeutic targets. Arch Dermatol Res.

[18] Diaz A (2020). Keloid lesions show increased IL-4/IL-13 signaling and respond to Th2-targeting dupilumab therapy. J Eur Acad Dermatol Venereol.

[19] Li X (2017). Status of M1 and M2 type macrophages in keloid. Int J Clin Exp Pathol.

[20] Hesketh M (2017). Macrophage phenotypes regulate scar formation and chronic wound healing. Int J Mol Sci.

[21] Kim TG (2018). Skin-specific CD301b^+^ dermal dendritic cells drive IL-17-mediated psoriasis-like immune response in mice. J Invest Dermatol.

[22] Lucas T (2010). Differential roles of macrophages in diverse phases of skin repair. J Immunol.

[23] Shook BA (2018). Myofibroblast proliferation and heterogeneity are supported by macrophages during skin repair. Science.

[24] Douglass A (2008). Antibody-targeted myofibroblast apoptosis reduces fibrosis during sustained liver injury. J Hepatol.

[25] Lagares D (2017). Targeted apoptosis of myofibroblasts with the BH3 mimetic ABT-263 reverses established fibrosis. Sci Transl Med.

[26] Plikus M (2017). Regeneration of fat cells from myofibroblasts during wound healing. Science.

[27] Atcha H (2021). Mechanically activated ion channel Piezo1 modulates macrophage polarization and stiffness sensing. Nat Commun.

[28] Ud-Din S, Bayat A (2017). Non-animal models of wound healing in cutaneous repair: In silico, in vitro, ex vivo, and in vivo models of wounds and scars in human skin. Wound Repair Regen.

[29] Van den Broek LJ (2014). Human hypertrophic and keloid scar models: principles, limitations and future challenges from a tissue engineering perspective. Exp Dermatol.

[30] Zheng LS (2017). SPINK6 promotes metastasis of nasopharyngeal carcinoma via binding and activation of epithelial growth factor receptor. Cancer Res.

