# Supplementary material for: IL-13RA2 downregulation in fibroblasts promotes keloid fibrosis via JAK/STAT6 activation
Source: JCI Insight. 2023 Mar 22;8(6):e157091. doi: 10.1172/jci.insight.157091 (PMC10070111; doi:10.1172/jci.insight.157091)

Supplementary Information

Supplementary Fig. 1–9

Table S1. Clinical characteristics of keloid patients.

Table S2. List of reagents.

Table S3. List of mRNA primer sequences used in this study.

Table S4. List of si-IL13RA2 sequences.

Supplementary Figure Legend

Supplementary Figure 1. Masson staining of normal skin and keloid. Scale bar, 100μm.

Supplementary Figure 2. Concentration of IL13 of indicated co-cultured system.

Supplementary Figure 3. Cell cycle assay by PI staining after IL13RA2 overexpression in KF.

Supplementary Figure 4. Western blotting of NFb-NC, NFb-si-1 and NFb-si-3.

Supplementary Figure 5. Western blotting of KF treated with indicated concentration of AS1517499.

Supplementary Figure 6. Concentration of IL13 of co-cultured system that KFs were pre-treated with or without 800nM AS1517499.

Supplementary Figure 7. IHC staining of IL13RA2 of patient biopsy that used for animal model. Scale bar, 100μm.

Supplementary Figure 8. TUNEL staining of Day0 implantations in papillary and reticular dermis(n=2). Scale bar, 100μm.

Supplementary Figure 9. Bubble plots of GO and KEGG pathway enrichment for down-regulated genes.

Supplementary Table 1

**Sex**	**Age**	**Diagnosis**	**Primary fibroblast**
Male	43y	Scar contracture skin graft (clavicular epithelial removal)	NFb
Male	26y	Precordial keloid	03KF
Female	38y	Precordial keloid	04KF
Male	38y	Precordial keloid	05KF
Female	54y	Umbilical keloid	
Female	25y	Precordial keloid	07KF
Female	1y	Polydactyly	08NF

Female	36y	Mammary hypertrophy	10NF
Female	25y	Earlobe keloid	11KF
Female	53y	Precordial keloid	12KF
Female	24y	Earlobe keloid	13KF
Female	28y	Earlobe keloid	14KF
Female	19y	Scapula keloid(multiple)	15KF
Female	1y	Polydactyly	16NF
Female	29y	Precordial keloid	17KF
Female	37y	Precordial keloid	18KF
Male	27y	Precordial keloid	
Male	46y	Precordial keloid	19KF
Female	52y	Lateral chest wall keloid	20KF
Female	19y	Precordial keloid	
Female	32y	Precordial keloid	
Female	51y	Precordial keloid	23KF
Female	33y	Precordial keloid	24KF
Male	23y	Dorsum keloid（multiple, recrudescence）	27KF
Male	35y	Precordial keloid	28KF
Female	33y	Precordial keloid	29KF
Female	43y	Scar contracture (abdominal skin graft)	NF1
Female	42y	Plantar melanoma (abdominal skin graft)	NF2
Male	40y	Occiput lipoma	NF3
Male	43y	Precordial keloid	Balb/C keloid implantation model

Supplementary Table 2

**Product**	**Cat#**	**Company**
Ki67 antibody	ab15580	Abcam
CD31 antibody	77699	Cell Signaling Technology
CD68 antibody	76437	Cell Signaling Technology
CD163 antibody	93498	Cell Signaling Technology
IL13RA2 antibody	85677	Cell Signaling Technology
FN1antibody	610077	BD Bioscience
COL1A1 antibody	72026	Cell Signaling Technology
COL3A1 antibody	66887	Cell Signaling Technology

pSTAT6 antibody	6391(Western blotting)	Cell Signaling Technology
	Ab263947 (IHC)	Abcam
STAT6 antibody	5397	Cell Signaling Technology
MMP9 antibody	1379008	Affinity
MMP2 antibody	40994	Cell Signaling Technology
a-Tubulin antibody	3873	Cell Signaling Technology
vimentin antibody	5741	Cell Signaling Technology
a-SMA antibody	ab124964	Abcam
GAPDH antibody	100080	Proteintech
BCL2L1 antibody	2764	Cell Signaling Technology
BCL2 antibody	15071	Cell Signaling Technology
Goat Anti-Rabbit IgG H&L (Alexa Fluor® 594)	ab150080	Abcam
DAPI	62247	ThermoFisher
AS1517499	S8685	Selleck
IL13	10369-HNAC	Sino Biological
INTERFERin	409-10	Polyplus
CCK8	EZB-CK8	EZBioscience
Annexin V-APC/7-AAD Apoptosis Detection Kit	KGA1023	KeyGEN
TUNEL staining kit	E-CK-A320	Elabscience
Masson staining kit	G1006	Servicebio
RNALater	AM7024	ThermoFisher
Tissue RNA Extraction Kit	EZB-RN001-plus	EZBioscience
Express RNA Extraction Kit	B0004DP	EZBioscience
Color Reverse Transcriptase Kit	A0010CGQ	EZBioscience
2 color X SYBR Green qPCR Mix	A0012-R2	EZBioscience
RIPA	P0013B	Beyotime Biotechnology
Protease inhibitor	05056489001	Roche

phosphatase inhibitors	04906837001	Roche
Pierce™ BCA Protein Assay Kit	23227	ThermoFisher
5×SDS-PAGE Protein Loading Buffer	20315ES05	Yeasen
collagenase I	MB2686	Meilunbio
PMA	P8139	Sigma Aldrich

Supplementary Table 3

Primer Name	Primer Sequence
GAPDH-Forward	GTCTCCTCTGACTTCAACAGCG
GAPDH-Reverse	ACCACCCTGTTGCTGTAGCCAA
IL4R-Forward	CTGCTCATGGATGACGTGGTCA
IL4R-Reverse	GGTGTGAACTGTCAGGTTTCCTG
IL13RA1-Forward	CCTGAATGAGAGGATTTGTCTGC
IL13RA1-Reverse	CAGTCACAGCAGACTCAGGATC
IL13RA2-Forward	GTGGAGTGATAAACAATGCTGGG
IL13RA2-Reverse	TGGGTAGGTGTTTGGCTTACGC
IL-4-Forward	CCGTAACAGACATCTTTGCTGCC
IL-4-Reverse	GAGTGTCCTTCTCATGGTGGCT
IL-13-Forward	ACGGTCATTGCTCTCACTTGCC
IL-13-Reverse	CTGTCAGGTTGATGCTCCATACC
SOCS1-Forward	TTCGCCCTTAGCGTGAAGATGG
SOCS1-Reversed	TAGTGCTCCAGCAGCTCGAAGA
SOCS3-Forward	CATCTCTGTCGGAAGACCGTCA
SOCS3-Reversed	GCATCGTACTGGTCCAGGAACT
MMP9-Forward	GCCACTACTGTGCCTTTGAGTC
MMP9-Reversed	CCCTCAGAGAATCGCCAGTACT
Arg-1-Forward	TCATCTGGGTGGATGCTCACAC
Arg-1-Reversed	GAGAATCCTGGCACATCGGGAA

Supplementary Table 4

No.	Primer	Sequence(5'to3')
1	IL13RA2（human）siRNA-67	GCAAUAUCAAGGUUUUAAATT
		UUUAAAACCUUGAUAUUGCTT
2	IL13RA2（human）siRNA- 225	GGAUACUUAGGUUAUCUCUTT
		AGAGAUAACCUAAGUAUCCTT
3	IL13RA2（human）siRNA- 394	GCAUUGAAGCGAAGAUACATT
		UGUAUCUUCGCUUCAAUGCTT

Supplementary Figure 1

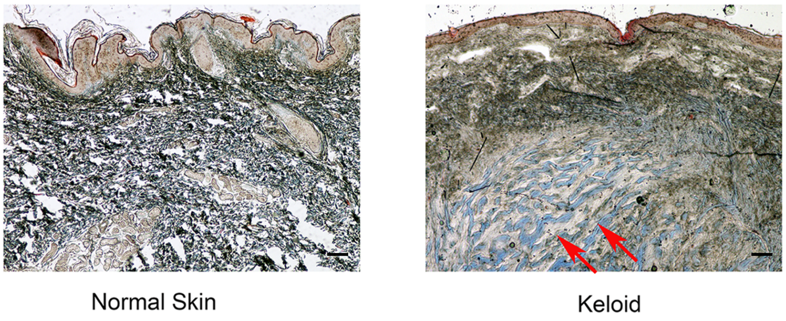

Supplementary Figure 2

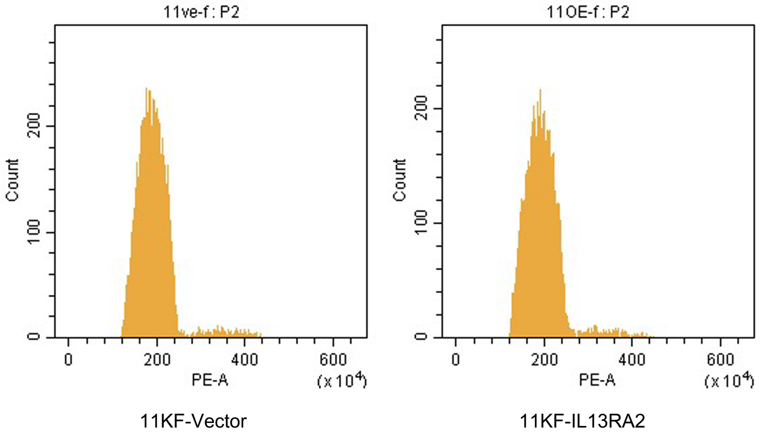

Supplementary Figure 3

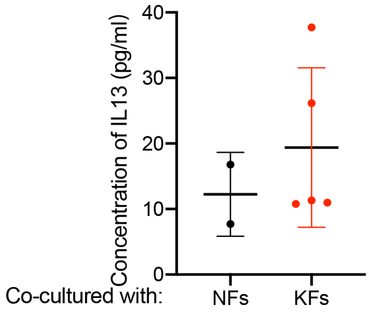

Supplementary Figure 4

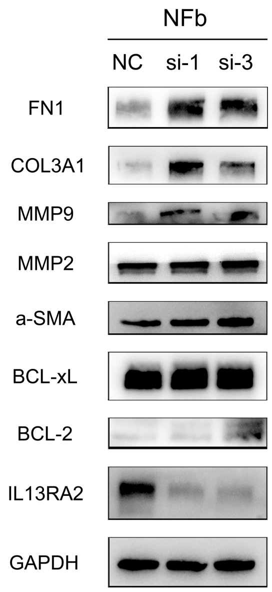

Supplementary Figure 5

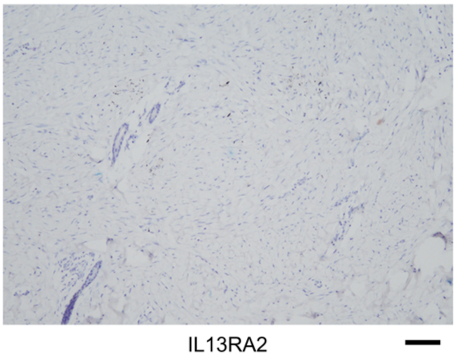

Supplementary Figure 6

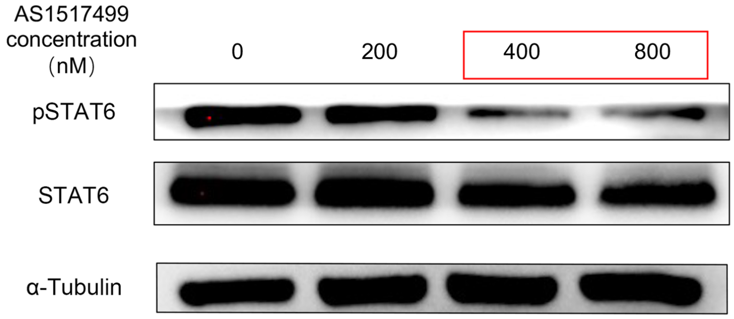

Supplementary Figure 7

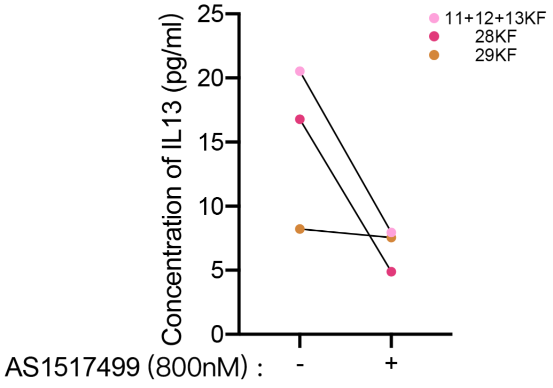

Supplementary Figure 8

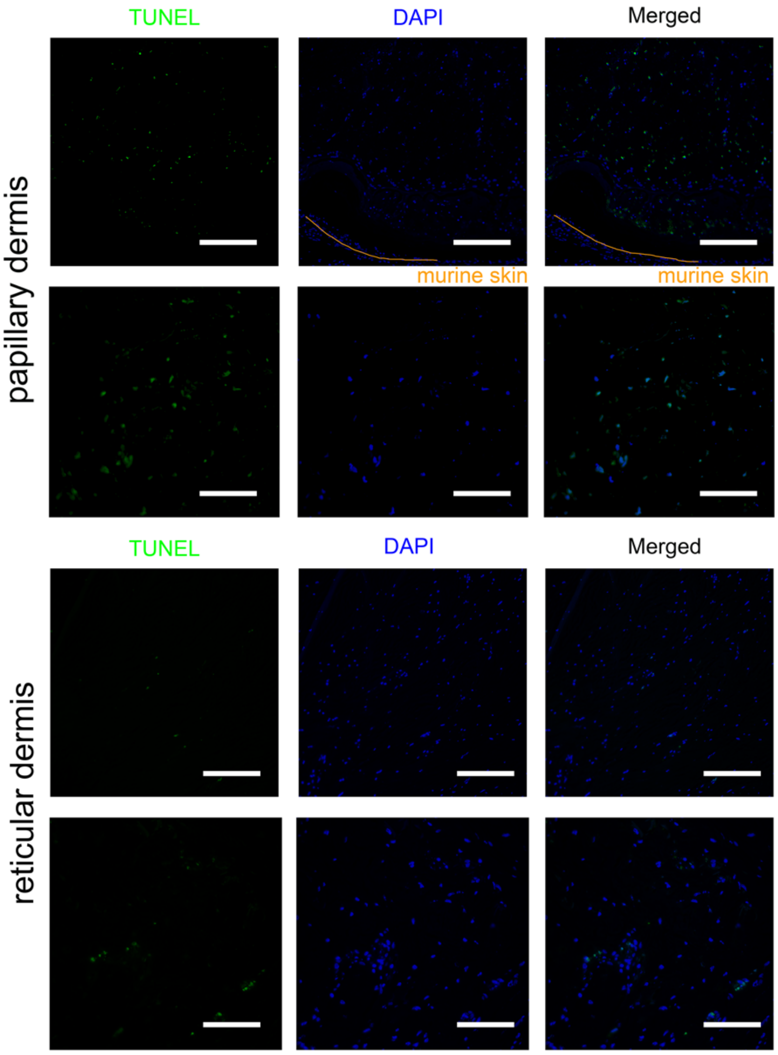

Supplementary Figure 9

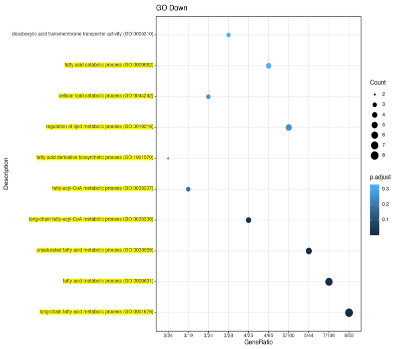
GO Down
dicarboxylic acid transmembrane transporter activity (GO:0005310)
fatty acid catabolic process (GO:0009062)
cellular lipid catabolic process (GO:0044242)
regulation of lipid metabolic process (GO:0019216)
fatty acid derivative biosynthetic process (GO:1901570)
fatty-acyl-CoA metabolic process (GO:0035337)
long-chain fatty-acyl-CoA metabolic process (GO:0035336)
unsaturated fatty acid metabolic process (GO:0033559)
fatty acid metabolic process (GO:0006631)
long-chain fatty acid metabolic process (GO:0001676)
Description
2/24
3/19
3/24
3/28
4/25
4/65
5/100
5/44
7/106
8/55
GeneRatio
Count
2
3
4
5
6
7
8
p.adjust
0.3
0.2
0.1

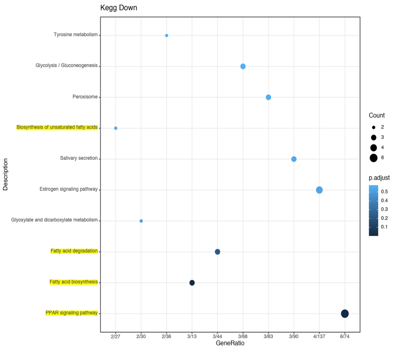
Kegg Down
Tyrosine metabolism
Glycolysis / Gluconeogenesis
Peroxisome
Biosynthesis of unsaturated fatty acids
Salivary secretion
Estrogen signaling pathway
Glyoxylate and dicarboxylate metabolism
Fatty acid degradation
Fatty acid biosynthesis
PPAR signaling pathway
Description
2/27
2/30
2/36
3/13
3/44
3/68
3/83
3/90
4/137
6/74
GeneRatio
Count
2
3
4
6
p.adjust
0.5
0.4
0.3
0.2
0.1

64

IL13RA2 Downregulation in Fibroblast Promotes Keloid Fibrosis via JAK/STAT6 Activation

Full uncut blots

Antibodies have been mentioned in Supplementary Table.2 .

Full unedited blots for figure 1E

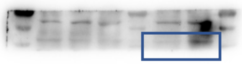
TGF-β

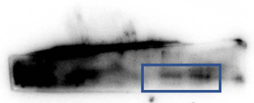
pSTAT6

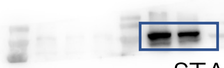
STAT6

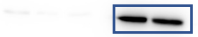
GAPDH

Full unedited blots for figure 2A

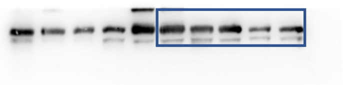

GAPDH

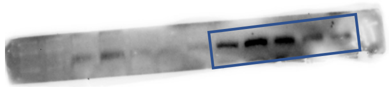

pSTAT6

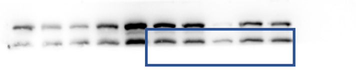

STAT6

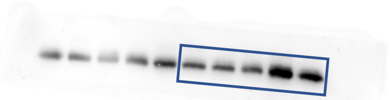

IL13RA2 (data not shown)

Full unedited blots for figure 2E

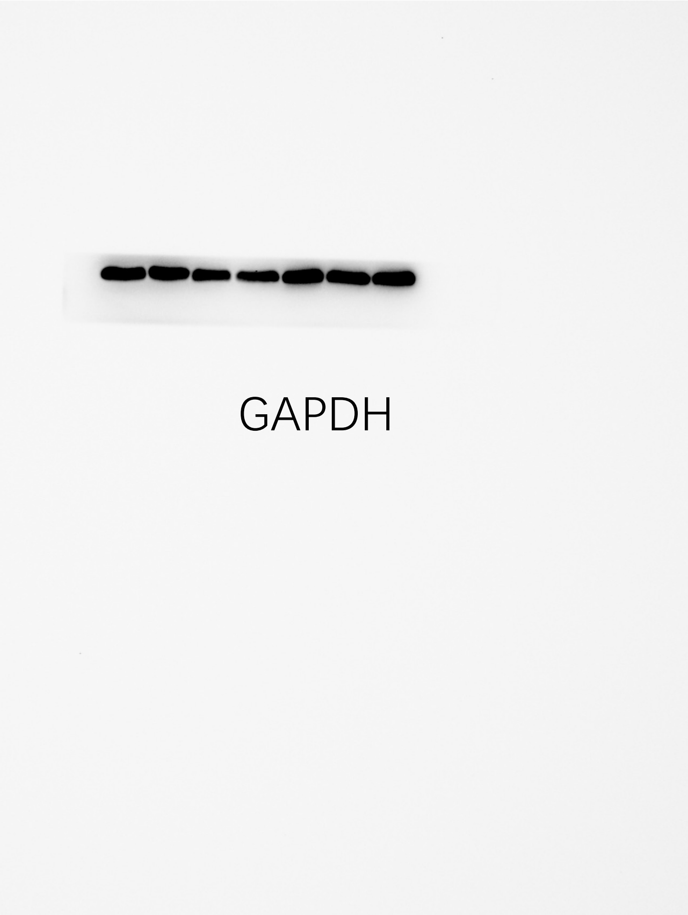

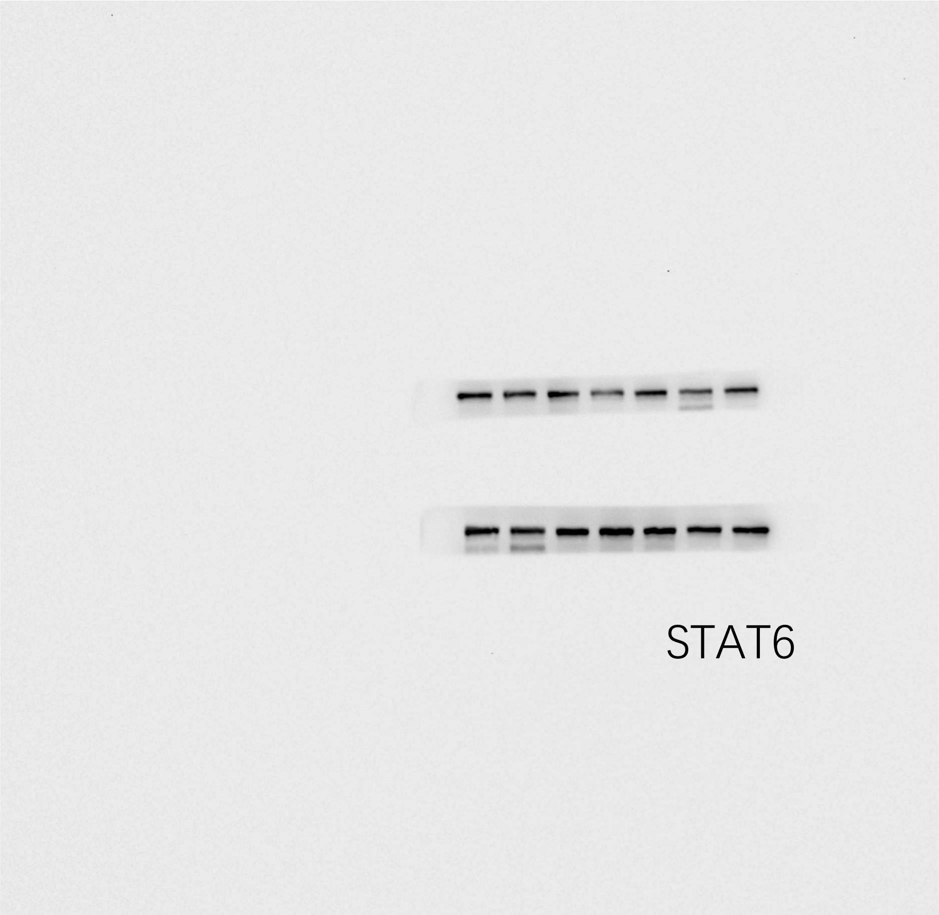

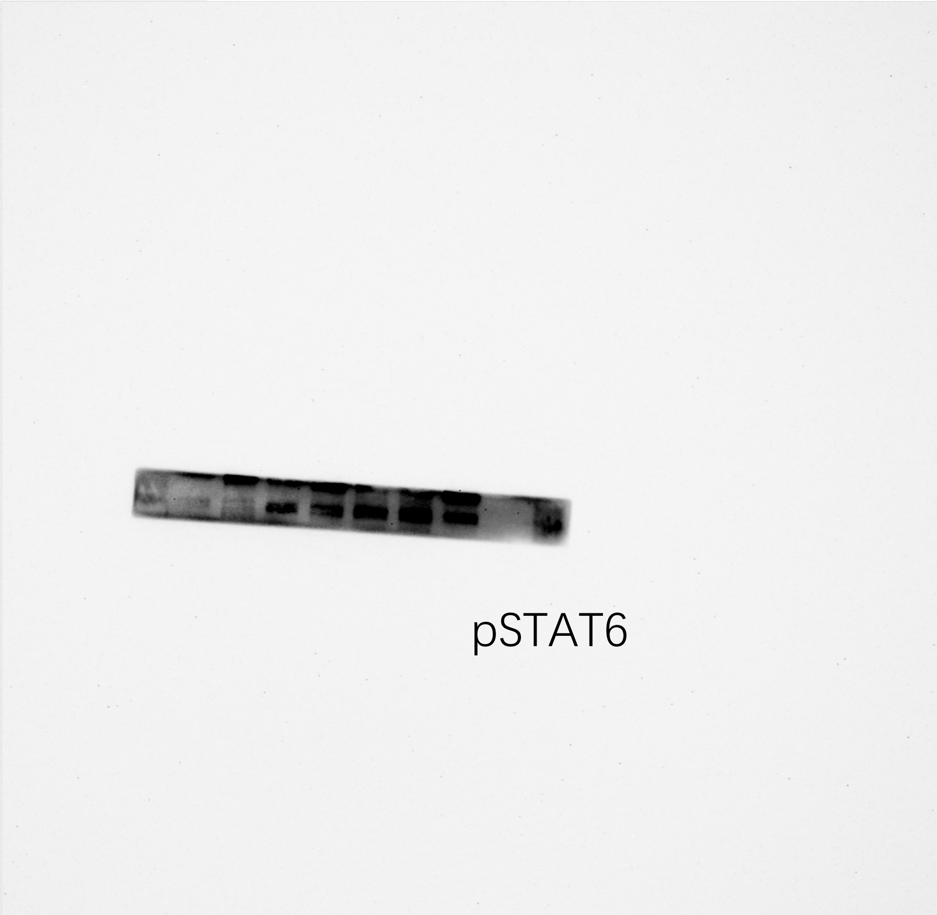

Full unedited blots for figure 3B

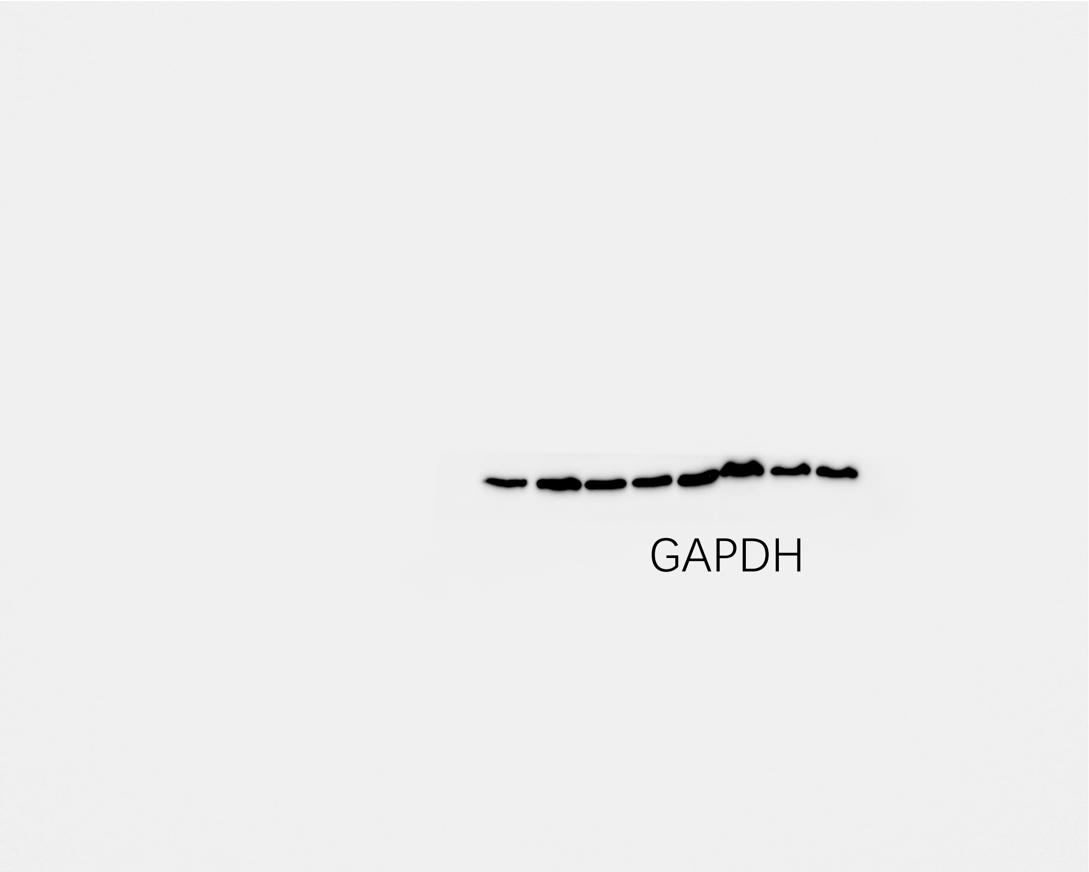

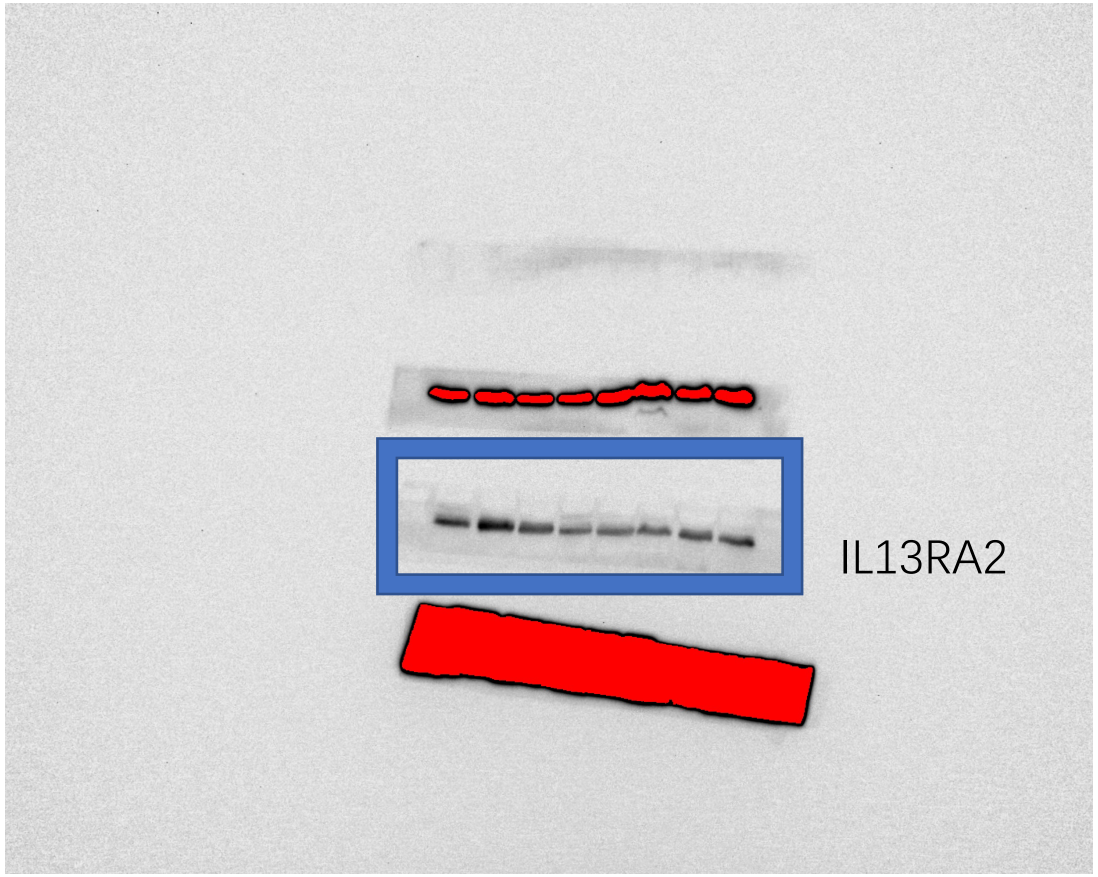

Full unedited blots for figure 3E

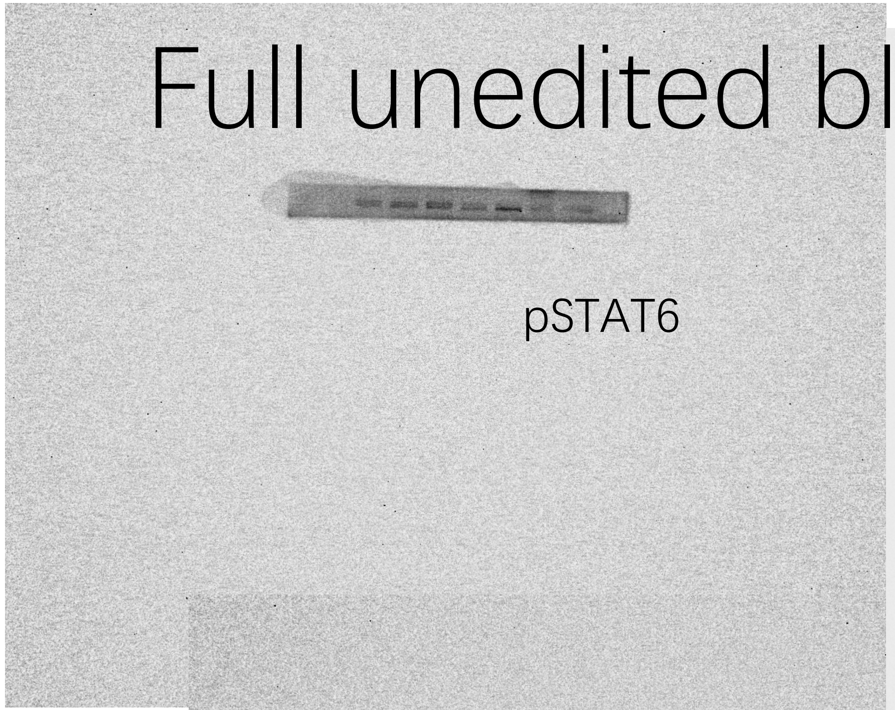

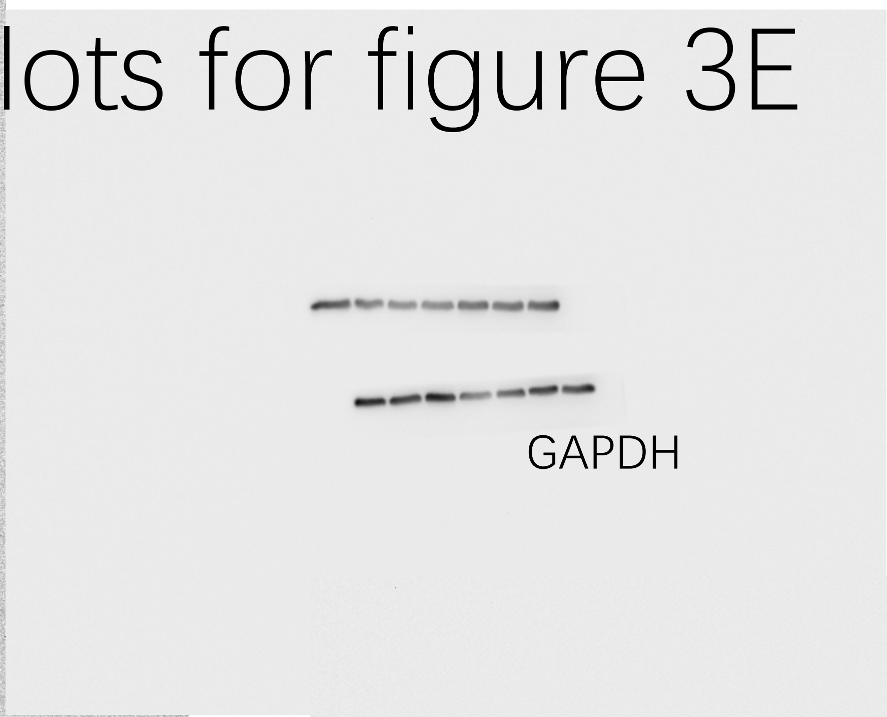

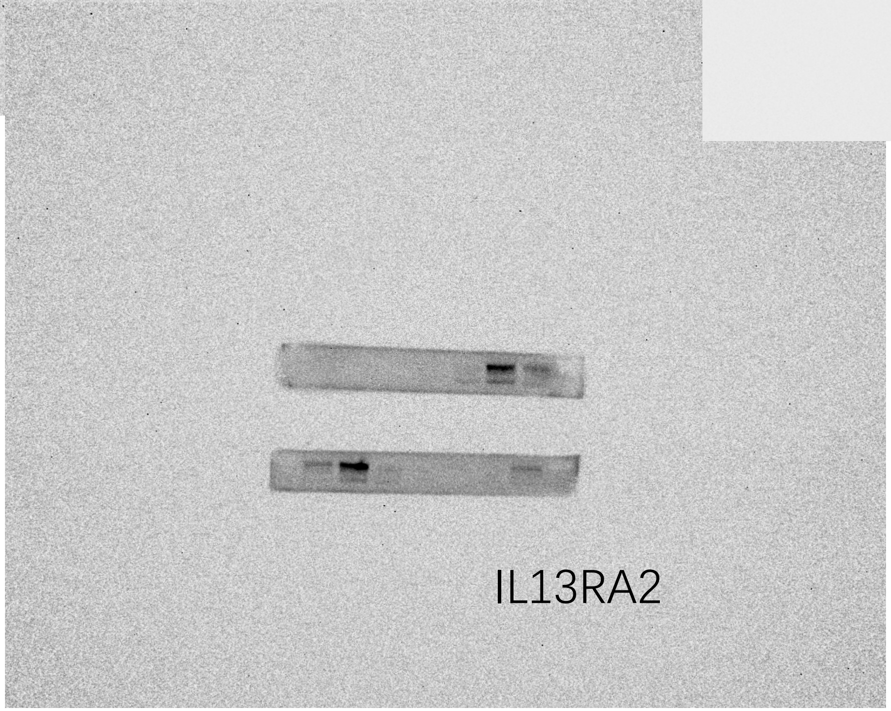

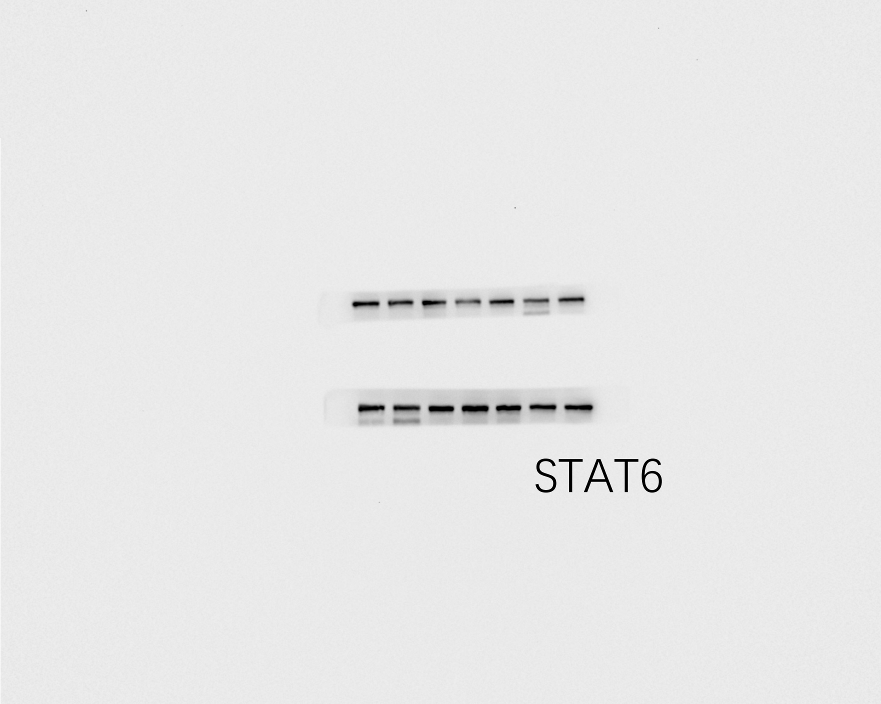

Full unedited blots for figure 4C

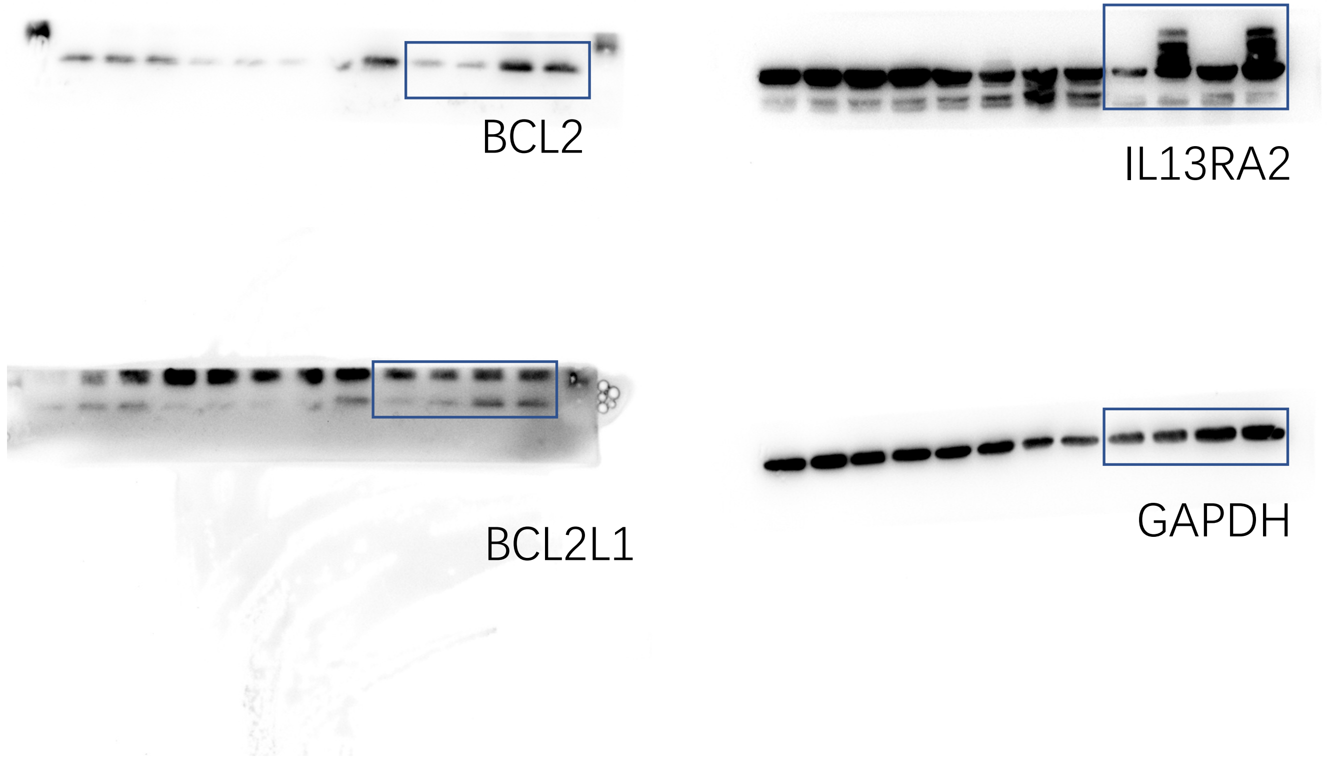

Full unedited blots for figure 4D

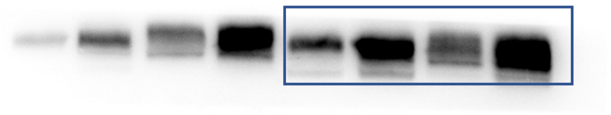

IL13RA2

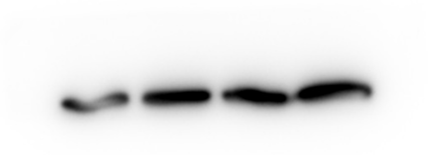

GAPDH

GAPDH of the same blot of IL13RA2

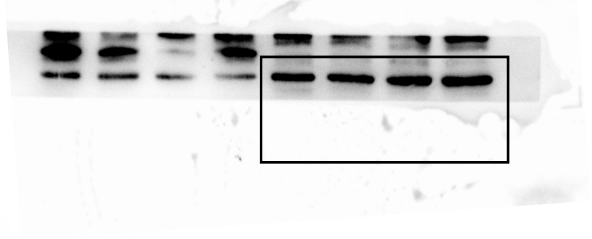

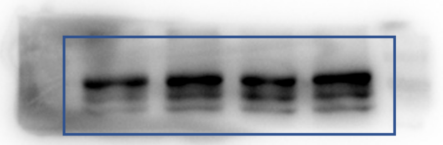

STAT6

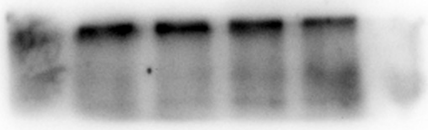

pSTAT6

Full unedited blots for figure 5C (20KF)

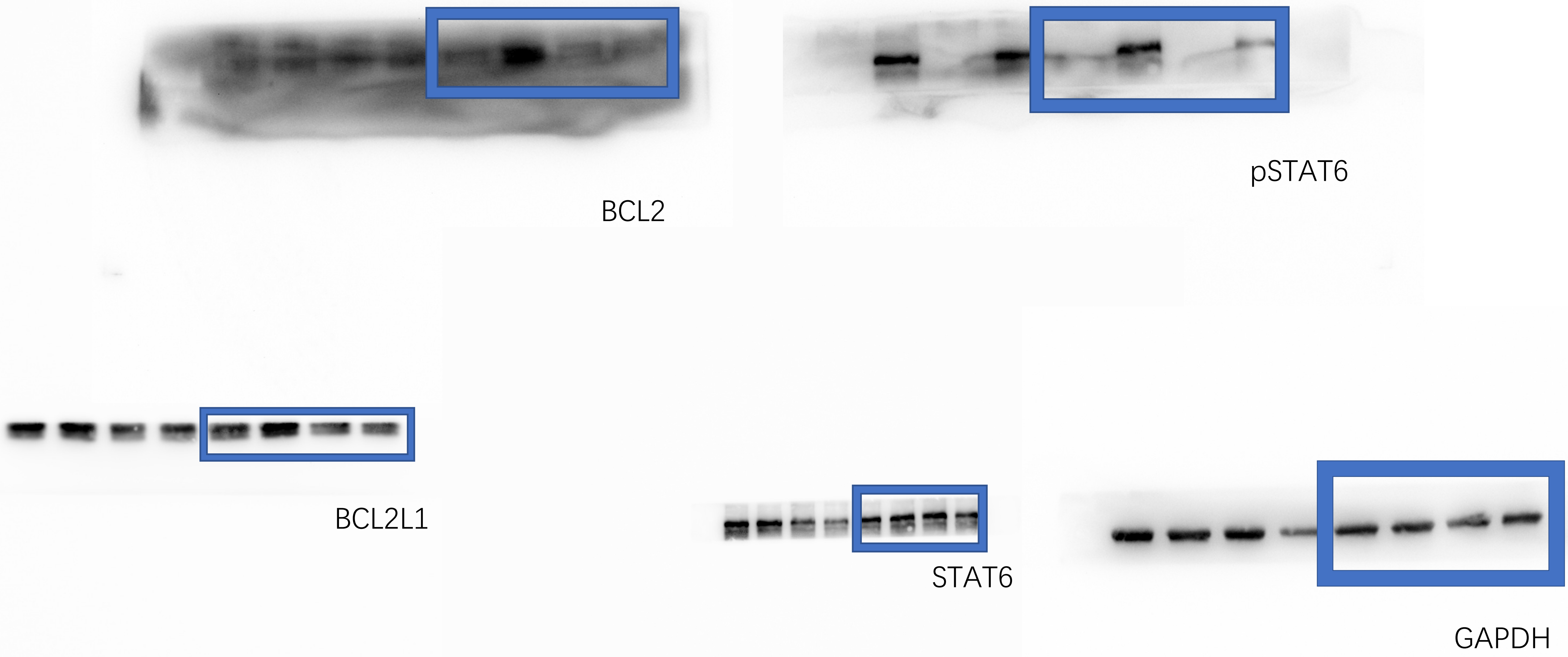

Full unedited blots for figure 5C (28KF)

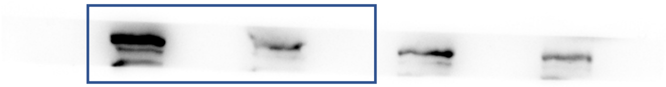

pSTAT6

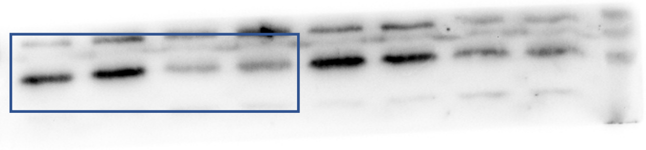

BCL2

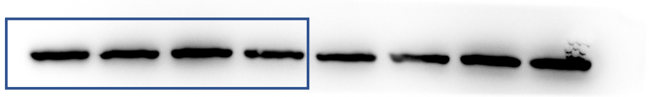

GAPDH

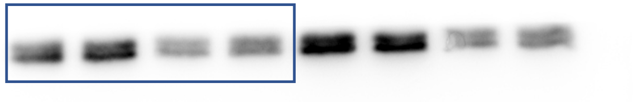

BCL2L1

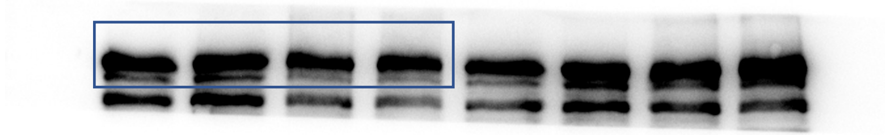

STAT6

Full unedited blots for figure 5F

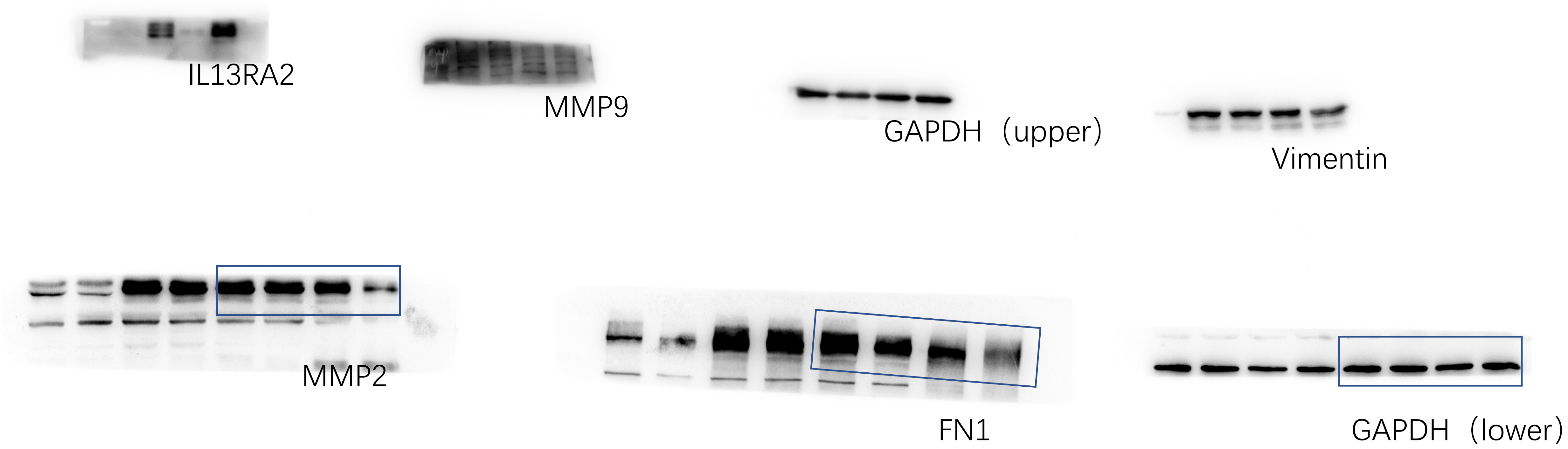

Full unedited blots for figure 5G

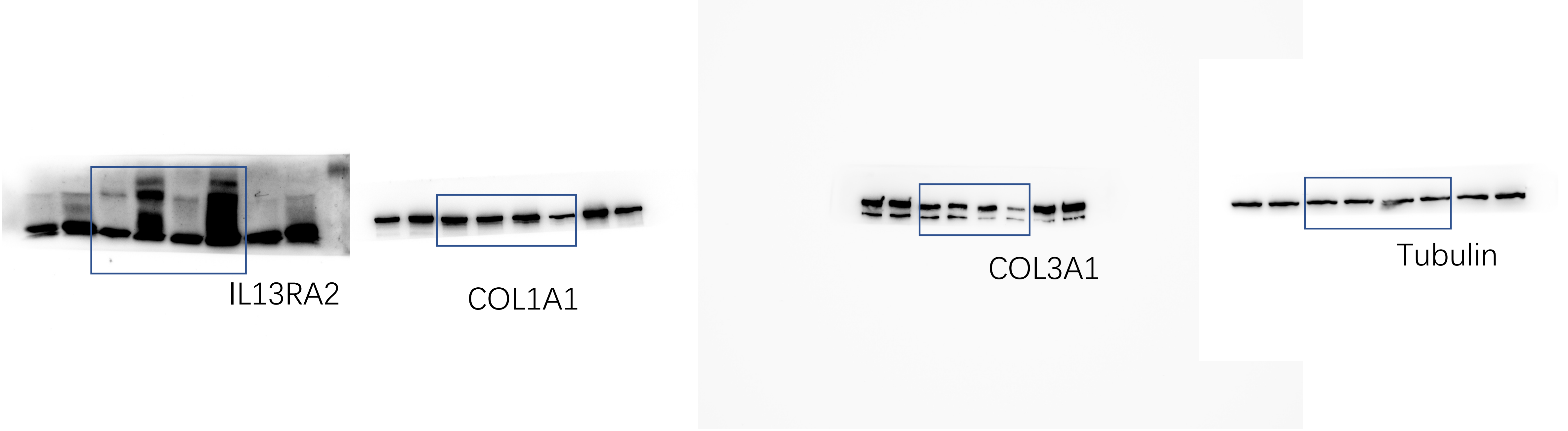

Full unedited blots for figure 5H

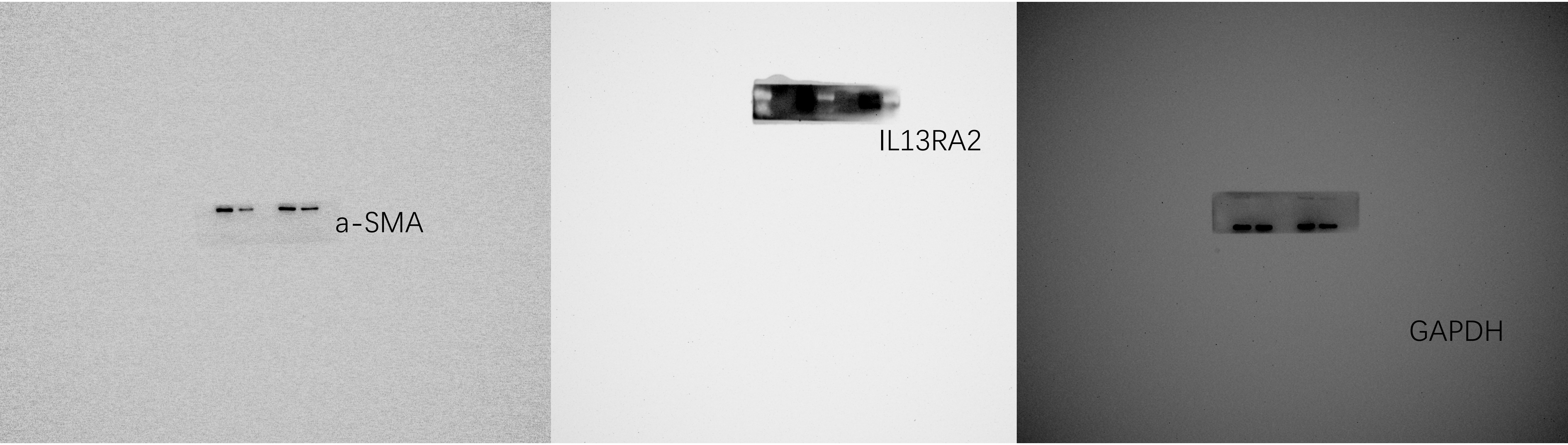

Full unedited blots for figure 6F

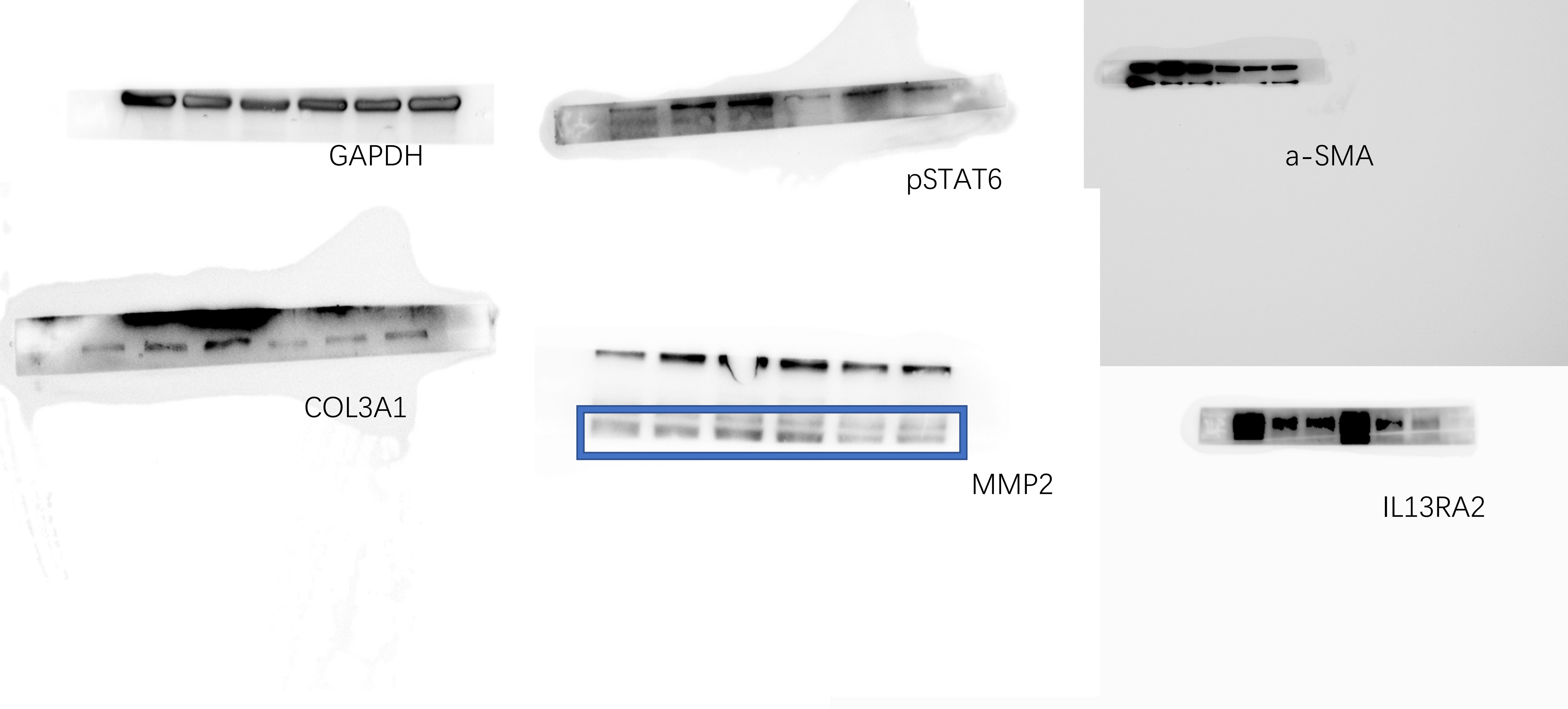

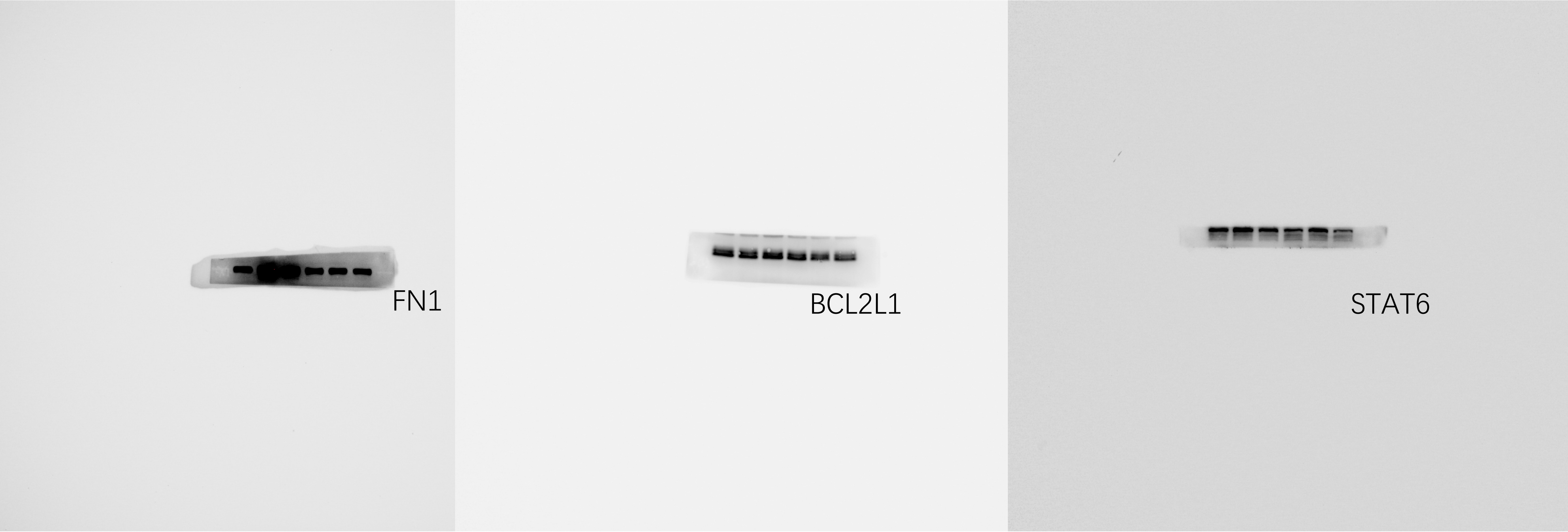
FN1
BCL2L1
STAT6

Full unedited blots for figure 7C

14KF
con 1uM
20KF
con 1uM

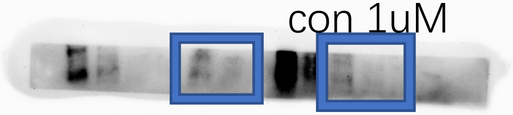

pSTAT6

14KF
con 1uM
20KF
con 1uM

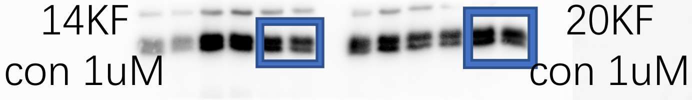

BCL2L1

14KF
con 1uM
20KF
con 1uM

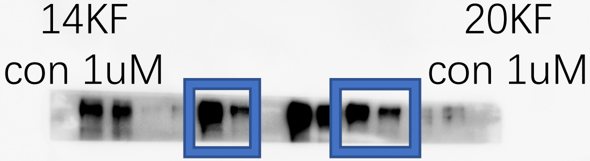

COL1A1

14KF
con 1uM
20KF
con 1uM

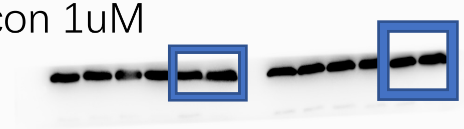

GAPDH

Loading control of BCL2L1

14KF
con 1uM
20KF
con 1uM

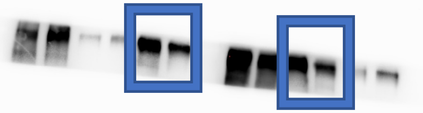

FN1

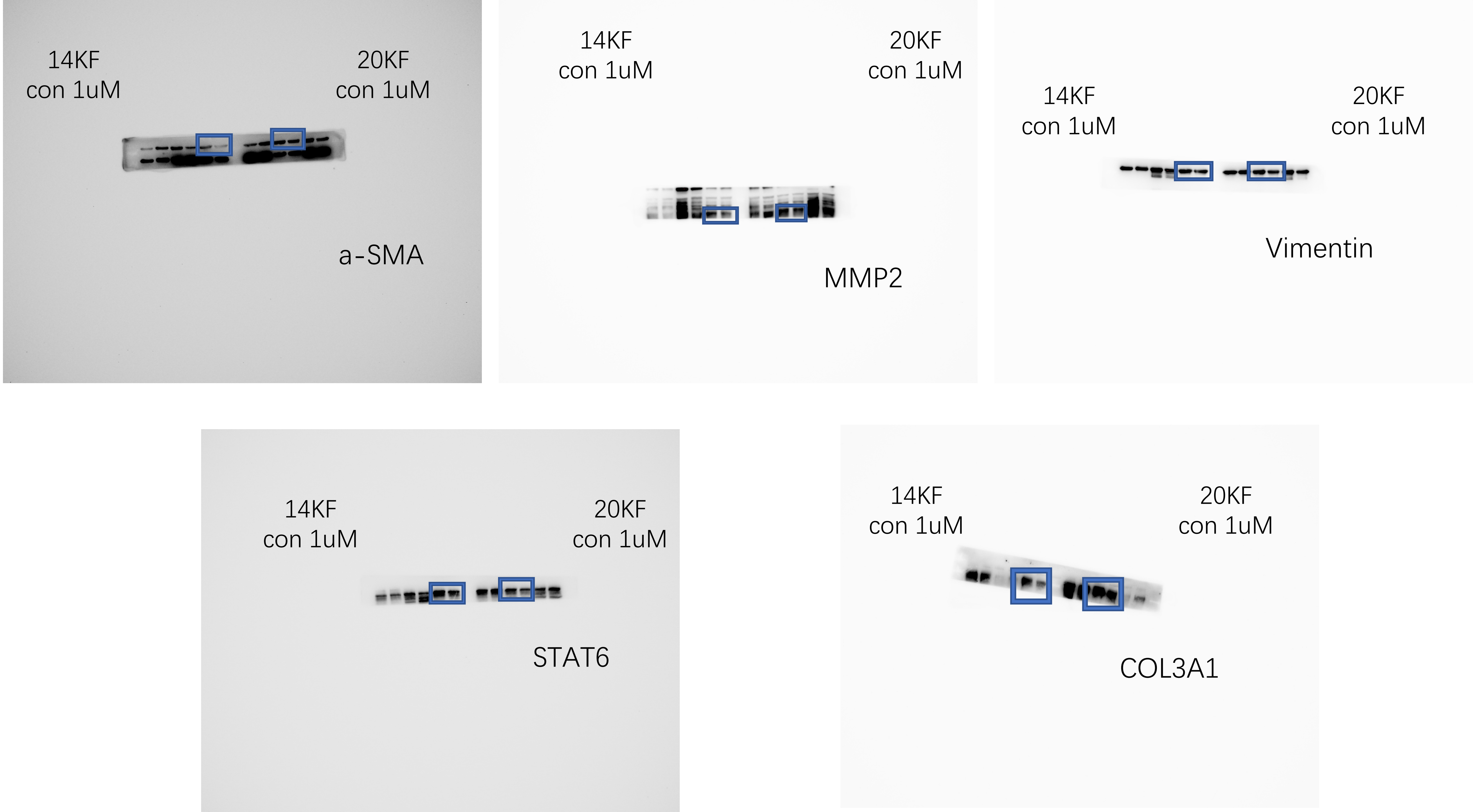
14KF
con 1uM
20KF
con 1uM
a-SMA
14KF
con 1uM
20KF
con 1uM
MMP2
14KF
con 1uM
20KF
con 1uM
Vimentin
14KF
con 1uM
20KF
con 1uM
STAT6
14KF
con 1uM
20KF
con 1uM
COL3A1

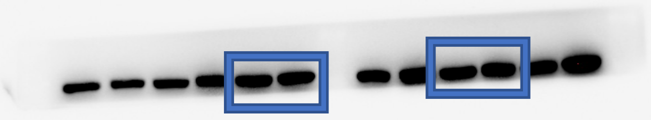

Tubulin (Loading control of figure 7c, except for BCL2L1)

Full unedited blots for figure 7D

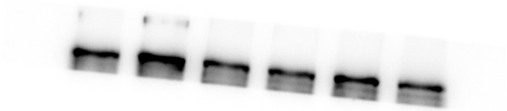

STAT6

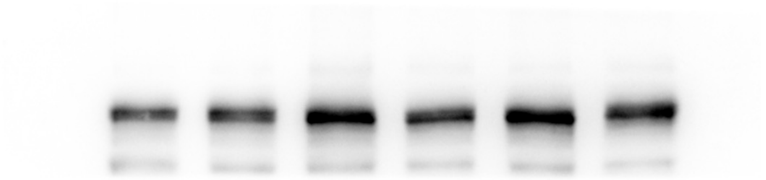

pSTAT6

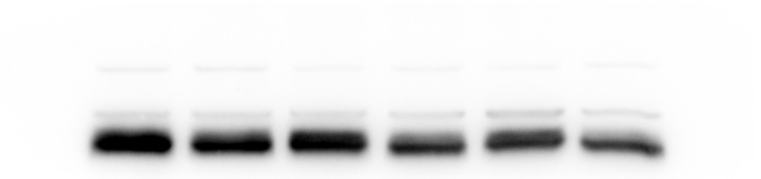

TGF- β

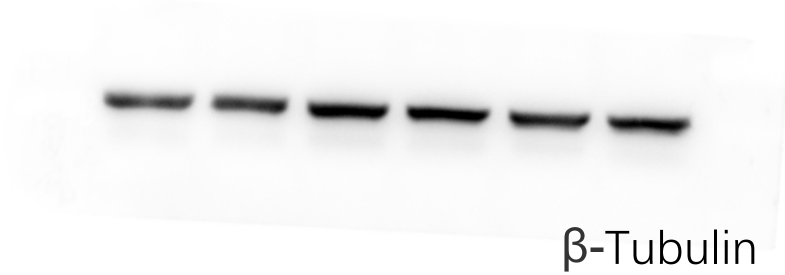

β-Tubulin

Full unedited blots for figure s

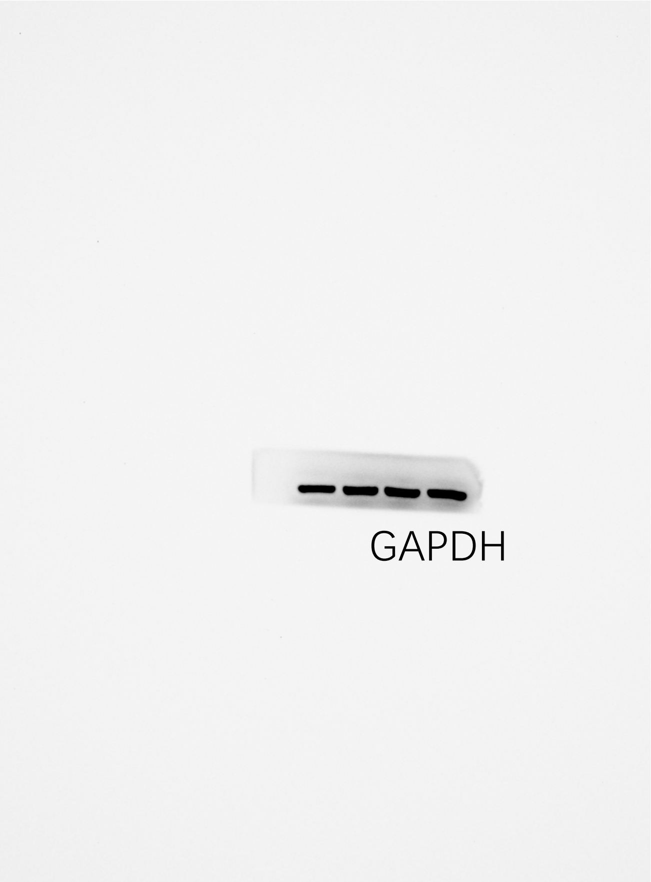

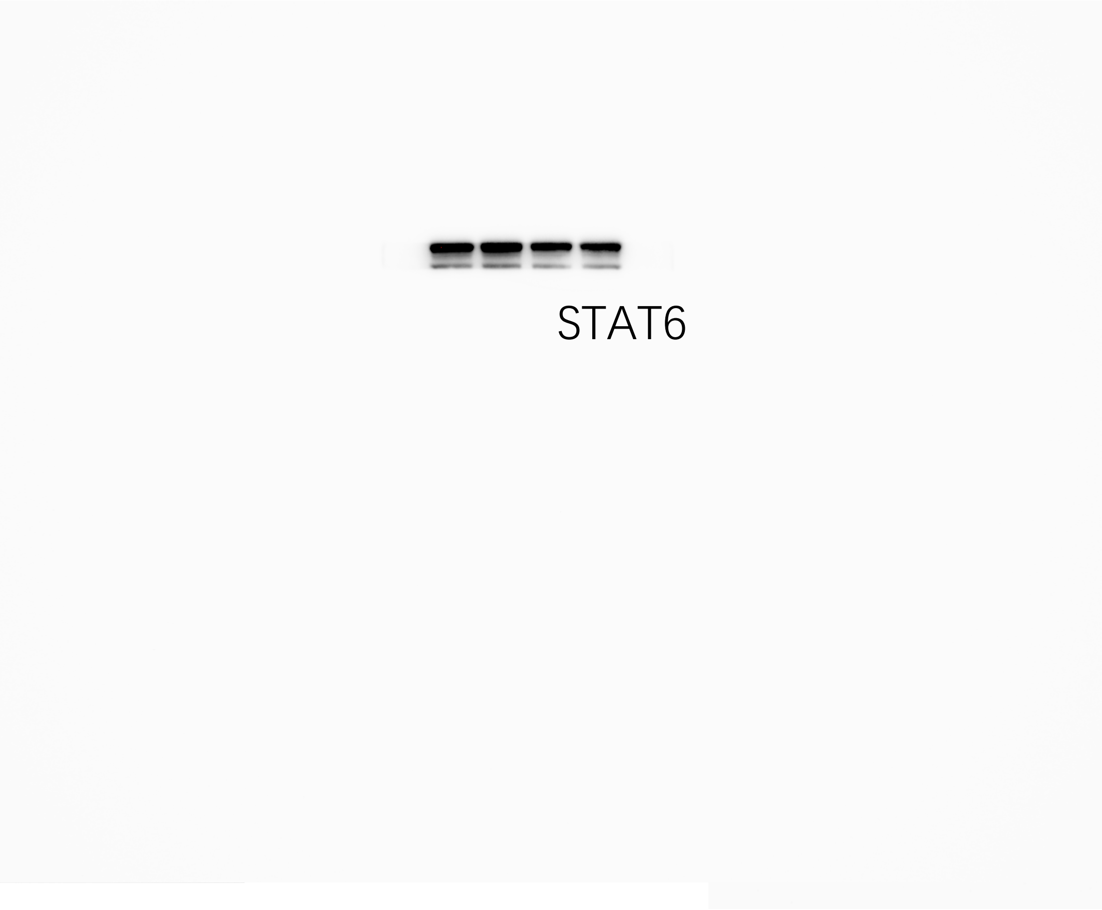

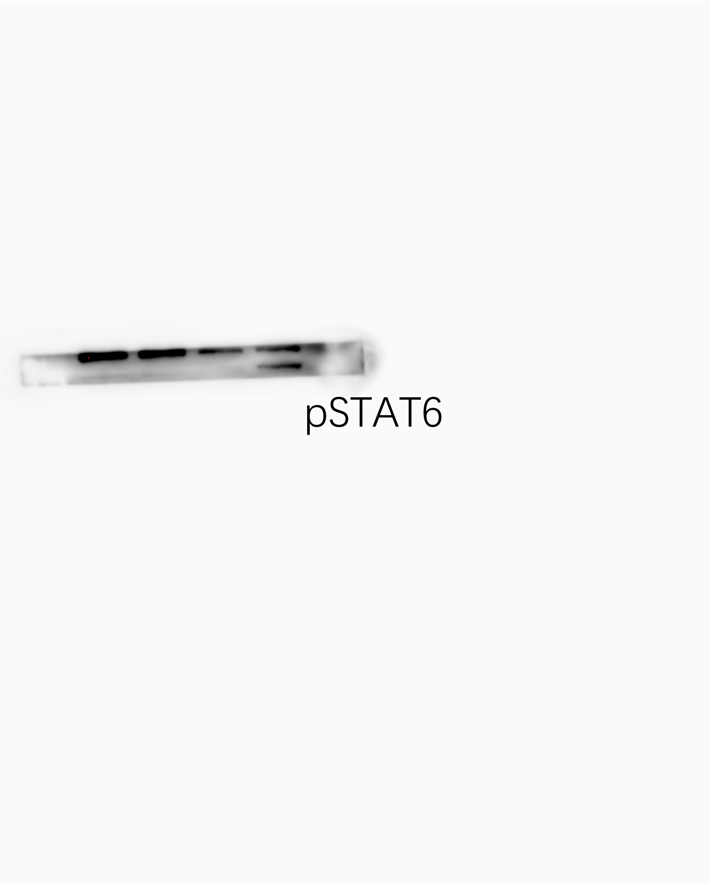

Supplement: Supplemental data [file jciinsight-8-157091-s062.pdf]
